# Levoketoconazole treatment in endogenous Cushing’s syndrome: extended evaluation of clinical, biochemical, and radiologic outcomes

**DOI:** 10.1530/EJE-22-0506

**Published:** 2022-10-17

**Authors:** Maria Fleseriu, Richard J Auchus, Yona Greenman, Sabina Zacharieva, Eliza B Geer, Roberto Salvatori, Rosario Pivonello, Ulla Feldt-Rasmussen, Laurence Kennedy, Michael Buchfelder, Beverly MK Biller, Fredric Cohen, Anthony P Heaney

**Affiliations:** 1 Oregon Health and Science University, Portland, OR, USA; 2 University of Michigan Medical School, Ann Arbor, MI, USA; 3 Tel Aviv University, Tel Aviv, Israel; 4 Medical University-Sofia, Sofia, Bulgaria; 5 Memorial Sloan Kettering Cancer Center, New York, NY, USA; 6 Johns Hopkins University, Baltimore, MD, USA; 7 Università Federico II di Napoli, Naples, Italy; 8 Rigshospitalet, Copenhagen University Hospital, Copenhagen, Denmark; 9 Cleveland Clinic, Cleveland, OH, USA; 10 University Hospital Erlangen, Erlangen, Germany; 11 Massachusetts General Hospital, Boston, MA, USA; 12 Xeris Biopharma Holdings, Inc., Chicago, IL, USA; 13 University of California Los Angeles School of Medicine, Los Angeles, CA, USA

## Abstract

**Objective:**

This extended evaluation (EE) of the SONICS study assessed the effects of levoketoconazole for an additional 6 months following open-label, 6-month maintenance treatment in endogenous Cushing’s syndrome.

**Design/Methods:**

SONICS included dose-titration (150–600 mg BID), 6-month maintenance, and 6-month EE phases. Exploratory efficacy assessments were performed at months 9 and 12 (relative to the start of maintenance). For pituitary MRI in patients with Cushing’s disease, a threshold of ≥2 mm denoted change from baseline in the largest tumor diameter.

**Results:**

Sixty patients entered EE at month 6; 61% (33/54 with data) exhibited normal mean urinary free cortisol (mUFC). At months 9 and 12, respectively, 55% (27/49) and 41% (18/44) of patients with data had normal mUFC. Mean fasting glucose, total and LDL-cholesterol, body weight, BMI, abdominal girth, hirsutism, CushingQoL, and Beck Depression Inventory-II scores improved from the study baseline at months 9 and 12. Forty-six patients completed month 12; four (6.7%) discontinued during EE due to adverse events. The most common adverse events in EE were arthralgia, headache, hypokalemia, and QT prolongation (6.7% each). No patient experienced alanine aminotransferase or aspartate aminotransferase >3× upper limit of normal, Fridericia-corrected QT interval >460 ms, or adrenal insufficiency during EE. Of 31 patients with tumor measurements at baseline and month 12 or follow-up, the largest tumor diameter was stable in 27 (87%) patients, decreased in one, and increased in three (largest increase 4 mm).

**Conclusion:**

In the first long-term levoketoconazole study, continued treatment through a 12-month maintenance period sustained the early clinical and biochemical benefits in most patients completing EE, without new adverse effects.

## Introduction

Endogenous Cushing’s syndrome (CS) is a rare, serious, endocrine disorder characterized by chronic overproduction of cortisol, most commonly due to a benign pituitary corticotroph tumor (i.e. Cushing’s disease (CD)) (1, 2, 3). CS is associated with significant metabolic, cardiovascular, musculoskeletal, and neuropsychiatric morbidities, increased mortality risk, and impaired quality of life (QoL) (4, 5, 6). Although surgery provides long-term remission in some patients, many may require lifelong medical therapy (1, 7). Therefore, long-term prospective clinical trial data are needed to inform the selection of medical therapies for patients with CS.

Levoketoconazole, the *2S, 4R* enantiomer of racemic ketoconazole, is an adrenal steroidogenesis inhibitor (8) approved in the United States for the treatment of endogenous hypercortisolemia in adult patients with CS for whom surgery is not an option or has not been curative (9). Preclinical and clinical pharmacology findings suggest that levoketoconazole is an approximately two-fold more potent cortisol synthesis inhibitor than ketoconazole, potentially accounting for essentially all of the cortisol synthesis inhibition activity of ketoconazole (10, 11). The short-term efficacy of oral levoketoconazole was established in two phase 3 multinational clinical studies: SONICS (Study Of levoketocoNazole In CS), a prospective, open-label, dose-titration and maintenance study (12), and LOGICS (LevOketoconazole to fill a Gap In CS), a double-blind, placebo-controlled, randomized withdrawal study (13). Here, we report findings from the extended evaluation (EE) phase of the SONICS study, which assessed, for the first time, longer-term effects of levoketoconazole on cortisol levels, biomarkers of CS comorbidities, clinical signs and symptoms of CS, and QoL. In addition, findings from pituitary adenoma imaging in the SONICS study are reported here.

## Methods

### Study design and patients

SONICS was a phase 3, multinational, single-arm, open-label study conducted in accordance with the ethical principles of the Declaration of Helsinki and the Good Clinical Practice Guideline of the International Conference on Harmonisation (ClinialTrials.gov: NCT01838551). The study protocol was approved by an institutional review board or independent ethics committee at each site (Supplementary Table 1), and all patients provided written informed consent to participate in the study.

SONICS included 3 phases: a flexibly timed (2–21 weeks) dose-titration phase, a maintenance phase to evaluate 6 months of treatment with a fixed therapeutic dose (primary endpoint), and an EE phase to assess an additional 6 months of levoketoconazole treatment (12). Inclusion and exclusion criteria have been previously reported (12); notably, patients with previous radiation therapy were eligible, provided treatment had occurred at least 4 years previously and no improvement was observed in the 6 months prior to the study. Patients who had been deemed surgical failures (≥6 weeks since surgery) were also eligible. Adults with a confirmed diagnosis of CS and 24-h mean urinary free cortisol (mUFC) levels of at least 1.5× the upper limit of normal (ULN) with either an abnormal dexamethasone suppression test or elevated late-night salivary cortisol (LNSC) were enrolled in the dose-titration phase, wherein the levoketoconazole dose was adjusted (within the range of 150−600 mg twice daily) based on mUFC response and tolerability. Patients for whom a therapeutic dose was identified were eligible to enter the maintenance phase. While a therapeutic goal, mUFC normalization was not required to advance to the maintenance phase if, in the opinion of the investigator, the participant derived clinical benefit from the study medication. There was no minimum duration of time during which the therapeutic dose was to be established. The therapeutic dose of levoketoconazole remained unchanged during the maintenance and EE phases unless adjustments were necessary to maintain cortisol control or to address safety or tolerability issues. Only patients completing the 6-month maintenance phase were invited to continue on to the 6-month EE phase reported here. Thus, patients could receive up to 12 months of maintenance treatment with levoketoconazole. EE phase assessments were scheduled at months 9 and 12 (relative to the start of maintenance).

### Outcomes

The exploratory outcomes evaluated in the EE phase and reported here included the proportion of patients with normal mUFC and changes from study baseline in mUFC, LNSC, and random serum cortisol concentrations. In addition, changes in several biomarkers and clinical parameters of CS comorbidities such as fasting glucose, HbA1c, total and LDL cholesterol, body weight, BMI, abdominal girth, blood pressure, C-reactive protein, investigator-assessed clinical signs and symptoms (e.g. acne, hirsutism (females only), peripheral edema), and patient-reported QoL (CushingQoL) and depression (Beck Depression Inventory-II (BDI-II)) were compared to study baseline (14, 15, 16, 17, 18, 19). Outcomes at months 6, 9, and 12 for the EE population, defined as patients who completed the dose-titration and maintenance phases and received at least 1 dose of levoketoconazole during EE (*n*  = 60), are summarized below and compared to the overall study safety population (*n* = 94) where appropriate.

mUFC was calculated from 2 to 4 (depending on visit) 24-h urine samples. LNSC was measured in saliva samples collected between 23:00 and 00:00 h on 1 or 2 nights. Serum cortisol was measured in blood samples collected at study visits, with no restrictions on time of day (i.e. random serum cortisol).

Safety assessments included monitoring of treatment-emergent adverse events (AEs), laboratory tests, electrocardiograms, and vital signs. Three classes of AEs were prespecified as being of special interest: hepatotoxicity, QT prolongation, and adrenal insufficiency (includes possible signs and symptoms of adrenal insufficiency). Disease-specific assessments included plasma adrenocorticotropic hormone (ACTH) and two-dimensional pituitary tumor size measurements from MRI scans centrally read by an experienced pituitary surgeon (MB). Treatment adherence (i.e. compliance, per protocol), defined as 80–120% medication intake of the dispensed dose, was determined from manual counts of returned study medication.

### Statistical analysis

Measures at study baseline and months 6, 9, and 12 of levoketoconazole treatment (relative to start of maintenance) were summarized using descriptive statistics. For all analyses reported here, ‘baseline’ refers to the main study baseline (before the first dose of levoketoconazole). Data for baseline and all on-treatment timepoints included only the EE population unless otherwise specified. Mean change values from study baseline at each timepoint were analyzed for statistical significance using paired *t*-tests without adjustment for multiplicity. Rates of mUFC normalization during the EE period were calculated based on observed data (without imputation of missing data) and regardless of study medication dose adjustments.

## Results

### Patients

**Figure 1:**
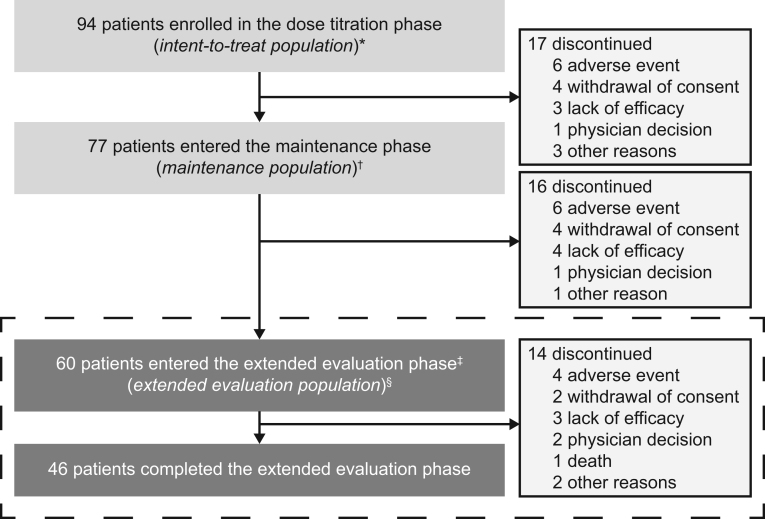
Patient disposition. *Intent-to-treat population included all patients who received ≥1 dose of levoketoconazole. ^†^Maintenance population consisted of all patients who entered the maintenance phase and received ≥1 dose of levoketoconazole during this phase. ^‡^One patient who completed the maintenance phase declined to enter the extended evaluation phase. ^§^Extended evaluation population consisted of all patients who entered the extended evaluation phase and received ≥1 dose of levoketoconazole during this phase. Results for the dose-titration and maintenance phases have been reported previously (12, 20, 21); the extended evaluation population is the subject of this report.

Of 94 enrolled patients, 61 (65%) completed the month 6 study visit, of whom 60 (98%) entered the EE phase (EE population; Fig. 1, Table 1). The majority (39/60 (65%)) of patients received levoketoconazole doses of 600 mg/day or less at the start of the EE period (Supplementary Table 2), with a median average daily dose of levoketoconazole during EE of 569 mg (range, 202–1190 mg). Forty-six (77%) patients in the EE population completed the study (month 12), representing 49% of the overall initial study population.

**Table 1:** Demographics and baseline characteristics (extended evaluation population). Data are presented as mean± s.d. or *n* (%).

Characteristics	Values
*n*	60
Age, years	43.8 ± 12.7
Range	19–72
Female sex	50 (83.3%)
White race	56 (93.3%)
Weight, kg	81.9 ± 23.4
Range	44–167
BMI, kg/m^2^	30.0 ± 7.3
Diagnosis of Cushing’s disease	51 (85.0%)
Diabetes	20 (33.3%)
Hypertension	42 (70.0%)
Pituitary adenoma*	
Microadenoma	27 (61.4%)
Macroadenoma	4 (9.1%)
Not visible on MRI	13 (29.5%)
mUFC at study baseline, nmol/24 h	
Mean ± s.d.	528.1 ± 438.8
Median (range)	394.7 (224.9–2776.9)
mUFC at study baseline, ×ULN^†^	
Mean ± s.d.	3.8 ± 3.2
Median (range)	2.9 (1.6–20.1)
Previous surgery	43 (71.7%)
Time since most recent surgery, months	
Mean ± s.d.	57.6±51.9
Median (range)	49.7 (2.3–233.2)
Prior radiotherapy	7 (11.7%)
Time since most recent radiotherapy, months	
Mean ± s.d.	113.6±69.0
Median (range)	88.9 (50.4–244.4)

*In the 44 patients in the extended evaluation population with Cushing’s disease and baseline MRI data. Pituitary adenoma size defined based on maximum tumor diameter (microadenoma <10 mm; macroadenoma ≥10 mm).

^†^ULN for UFC = 138 nmol/24 h.

mUFC, mean urinary free cortisol; UFC, urinary free cortisol; ULN, upper limit of normal.

### Efficacy

**Figure 2:**
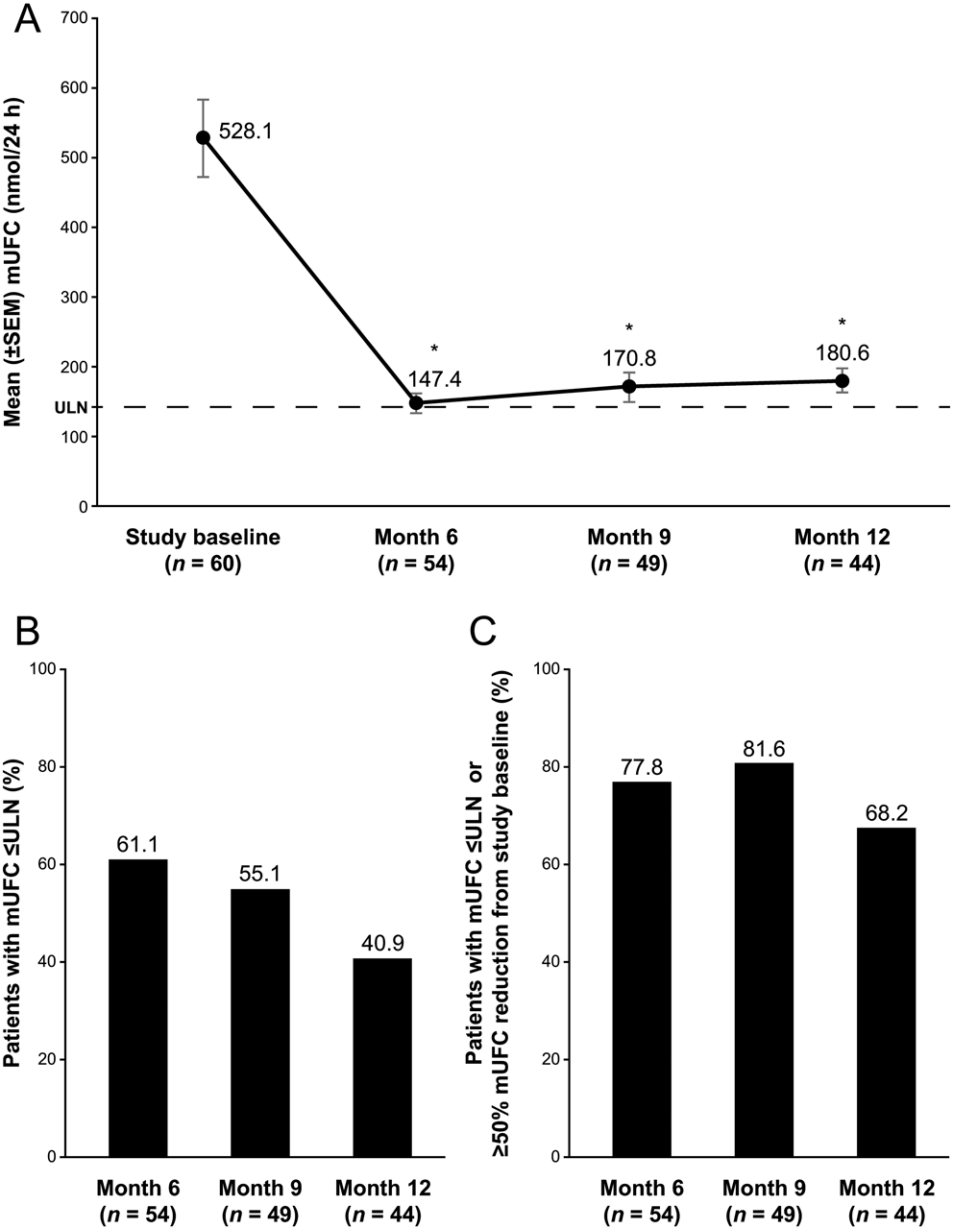
Mean mUFC concentrations and response over time (extended evaluation population). (A) Mean mUFC concentration at baseline, and months 6, 9, and 12, (B) mUFC response defined as normalization of mUFC, and (C) normalization of mUFC or ≥50% reduction from baseline. Dashed line represents the ULN for UFC (138 nmol/24 h). *P < 0.0001 vs baseline. mUFC, mean urinary free cortisol; UFC, urinary free cortisol; ULN, upper limit of normal.

The mean mUFC at study baseline was 528 nmol/24 h (191 µg/24 h (3.8× ULN)) in the EE population (Table 1). The mean decrease in mUFC was –398 nmol/24 h (–144 µg/24 h) at month 6, –384 nmol/24 h (–139 µg/24 h) at month 9, and –328 nmol/24 h (–119 µg/24 h) at month 12; all *P* < 0.0001 vs baseline (Fig. 2; Supplementary Tables 3 and 4). The proportion of patients with mUFC normalization was 61, 55, and 41% at months 6, 9, and 12, respectively, and the proportion of patients with mUFC normalization or at least a 50% reduction from baseline was 78% at month 6, 82% at month 9, and 68% at month 12. Using the denominator from the ITT population from the SONICS study (*n* = 94), the proportion of patients with mUFC normalization was 36, 29, and 19% at months 6, 9, and 12, respectively, and the proportion of patients with mUFC normalization or at least a 50% reduction from baseline was 46% at month 6, 43% at month 9, and 32% at month 12. All patients with drug accountability data and normal mUFC at month 12 (*n*  = 17) were adherent to treatment whereas 4 (15%) of 26 patients with above-normal mUFC at month 12 were not adherent (medication intake <80% of expected between months 9 and 12). Mean treatment adherence at month 12 was 93% of the expected dose among patients with mUFC above normal, as compared with 100% for patients with normal mUFC. The mean daily dose of levoketoconazole at months 9 and 12 was not statistically significantly different between responders and nonresponders (539.6 mg vs 536.2 mg at month 9 (*P* = 0.9695) and 605.8 mg vs 528.4 mg at month 12 (*P* = 0.4278)).

Of the nine enrolled patients in the SONICS study who had received prior radiation therapy, seven entered the EE phase, including one patient who was considered a responder in the primary endpoint analysis (i.e. mUFC normalization without a dose increase during maintenance phase). For the one responder, UFC levels rebounded and became abnormal following 2 weeks of levoketoconazole withdrawal at the end of the maintenance phase, suggesting that UFC normalization could not be attributed to radiation therapy. Of the seven patients who had received prior radiation therapy, three had mUFC in the normal range at month 6, four at month 9, and two at month 12 (Supplementary Table 5).

Mean baseline LNSC (*n*  = 58) was 12 nmol/L (ULN = 2.5 nmol/L), and mean baseline random serum cortisol (*n*  = 27) was 525 nmol/L. Mean decreases in LNSC and random serum cortisol (Supplementary Tables 3 and 4) were statistically significant relative to study baseline only for LNSC at month 6 (*P* = 0.028).

**Figure 3:**
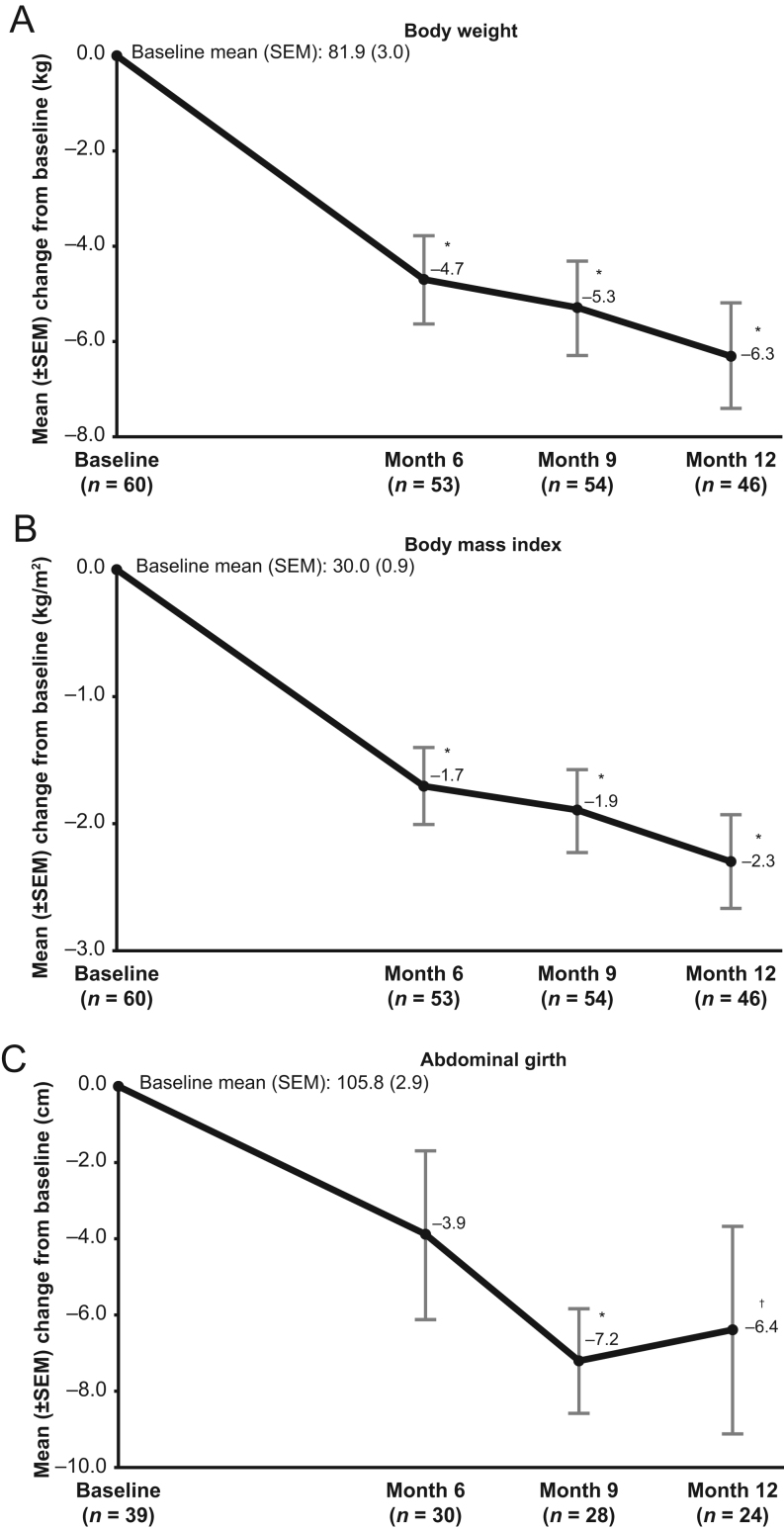
Mean changes from study baseline at months 6, 9, and 12 for (A) body weight, (B) BMI, and (C) abdominal girth (extended evaluation population). Two-sided *P* value from the paired *t*-test that was performed on the mean change from baseline to each timepoint. *P < 0.0001 vs baseline; ^†^ *P* < 0.05 vs baseline.

Reduction in mean values of CS comorbidity biomarkers observed at month 6 was generally maintained throughout the EE phase (Table 2; Fig. 3). The observed reductions from baseline at month 12 for fasting glucose; total, LDL, and HDL cholesterol; body weight; BMI; and abdominal girth were statistically significant. No significant mean changes from baseline in blood pressure or C-reactive protein were noted.

**Table 2:** Changes from study baseline in Cushing’s syndrome comorbidity biomarkers and clinical signs and symptoms (extended evaluation population).

Parameters	Study baseline		Change from study baseline to month 6		*P* value*	Change from study baseline to month 9		*P* value*	Change from study baseline to month 12		*P* value*
	Mean ± s.d.	*n*	Mean ± s.d.	*n*		Mean ± s.d.	*n*		Mean ± s.d.	*n*	
**Comorbidity biomarkers**											
Fasting glucose, mmol/L	5.7 ± 1.7	59	–0.6 ± 1.4	49	0.004	–0.4 ± 1.6	53	0.073	–0.4 ± 1.2	45	0.022
Hemoglobin A1c, %	6.0 ± 1.1	60	–0.4 ± 0.9	55	0.002	–0.3 ± 0.8	54	0.005	–0.3 ± 0.9	45	0.068
Total cholesterol, mmol/L	5.7 ± 1.2	58	–1.1 ± 1.0	52	<0.0001	–1.0 ± 1.1	52	<0.0001	–1.0 ± 0.9	43	<0.0001
LDL cholesterol, mmol/L	3.4 ± 1.2	58	–1.0 ± 0.9	52	<0.0001	–0.9 ± 0.9	52	<0.0001	–0.9 ± 0.8	43	<0.0001
HDL cholesterol, mmol/L	1.7 ± 0.5	58	–0.2 ± 0.4	52	<0.001	–0.1 ± 0.3	52	0.0001	–0.1 ± 0.3	43	0.007
**Clinical signs and symptoms†**											
Acne^‡^	2.5 ± 5.7	58	–1.9 ± 4.7	50	0.006	–2.0 ± 5.0	52	0.005	–1.4 ± 4.7	44	0.052
Hirsutism (females only)^§^	7.8 ± 6.1	49	–2.7 ± 4.8	43	<0.001	*–*3.3 ± 5.0	43	<0.0001	–4.0 ± 5.5	37	<0.0001
Peripheral edema^¶^	0.8 ± 1.6	58	–0.4 ± 1.3	48	0.030	–0.3 ± 1.4	52	0.074	–0.3 ± 1.6	43	0.189

*Two-sided *P* value from the paired *t*-test performed on the change from study baseline to months 6, 9, and 12.

^†^For all clinical signs and symptoms, a decrease from study baseline corresponds to improvement.

^‡^Based on acne global score: range from 0 to 44, where 0 = none, 1–18 = mild, 19–30 = moderate, 31–38 = severe, and ≥39 = very severe.

^§^Based on hirsutism total score: range from 0 (none) to 36 (worst).

^¶^Based on peripheral edema total score: range from 0 (none) to 12 (worst).

HDL, high-density lipoprotein; LDL, low-density lipoprotein.

**Figure 4:**
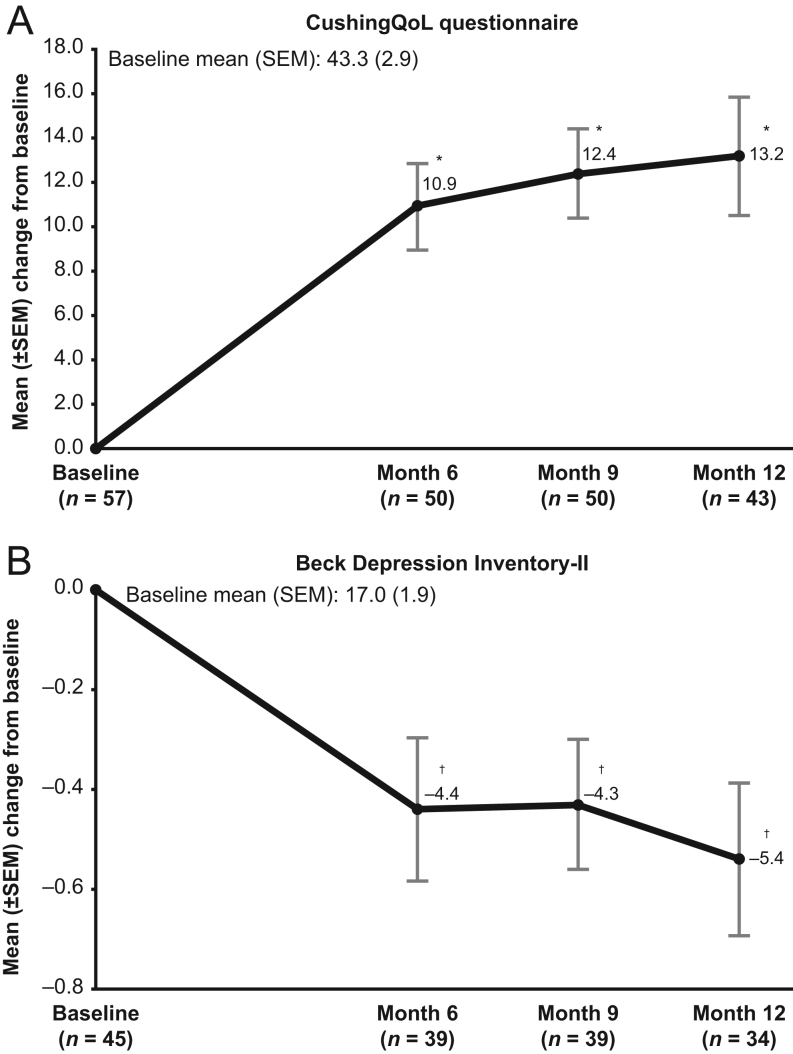
Mean changes from study baseline at months 6, 9, and 12 for (A) CushingQoL questionnaire and (B) BDI-II (extended evaluation population). CushingQoL questionnaire score: range from 0 (worst) to 100 (best); an increase from baseline corresponds to improvement. BDI-II score: range from 0 (best) to 63 (worst); a decrease from baseline corresponds to improvement. Two-sided *P* value from the paired *t*-test that was performed on the mean change from baseline to each timepoint. *P < 0.0001 vs baseline; ^†^ *P* < 0.01 vs baseline. BDI-II, Beck Depression Inventory-II.

In female patients, a significant mean decrease in hirsutism score was noted at all assessments through the end of the EE phase (Table 2). Significant mean decreases in acne scores were observed in the EE population at months 6 and 9. A mean decrease in peripheral edema score was statistically significant at month 6 only. Significant increases in mean CushingQoL scores and significant decreases in mean BDI-II scores (clinically meaningful improvement for both measures) (22, 23) were observed at months 6, 9, and 12 compared to baseline (Fig. 4).

### Adrenocorticotropic hormone levels and pituitary MRI

**Figure 5:**
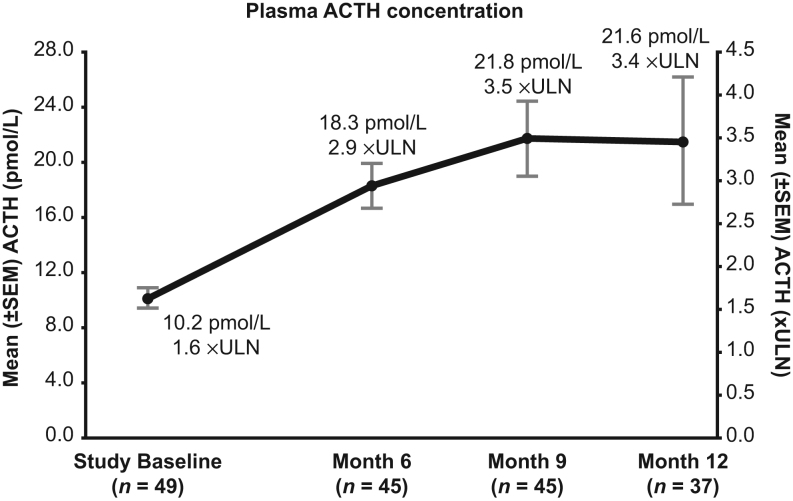
Mean ACTH concentrations in patients with Cushing’s disease at months 6, 9, and 12 (extended evaluation population). Values are shown in both pmol/L and xULN. ACTH ULN for males and females are 11.1 and 6 pmol/L, respectively. ACTH, adrenocorticotropic hormone; ULN, upper limit of normal.

In patients with CD, mean plasma ACTH increased significantly from baseline during EE (Fig. 5); however, there was no correlation at months 6, 9, or 12 between the change in ACTH concentration (calculated as fold-ULN) and change in mUFC (calculated as fold-ULN). Furthermore, the ACTH concentration appeared unrelated to mUFC normalization status at month 12 (Supplementary Table 6).

Forty-four of 51 patients with CD in the EE population had a baseline MRI, and 34 had a follow-up exam during EE, most commonly at month 12. At baseline, 4 (9.1%) patients had a macroadenoma and 27 (61%) had a microadenoma, with tumor not visible/measurable in 13 (30%). No tumors that were below the limit of detection on MRI became visible during therapy. During EE, 2 (5.9%) patients had a macroadenoma and 22 (65%) had a microadenoma. Using a threshold of ≥2 mm change in tumor diameter to denote size change, of 31 patients with tumor measurements at baseline and month 12 or follow-up, the largest diameter was stable in 27 (87%) patients, decreased in 1 patient (2 mm), and increased in 3 patients (largest increase 4 mm). No patients with an increase in tumor size seen on MRI discontinued levoketoconazole, or had a dose reduction, for that reason. Plasma ACTH and tumor size at month 12 were not correlated; changes from baseline in plasma ACTH and changes from baseline in tumor size at month 12 also were not correlated. There were no reported cases of Nelson’s syndrome or pituitary apoplexy, and no cases of visual changes suggestive of optic chiasm impingement.

### Safety

Safety findings during SONICS dose-titration and maintenance phases have been reported (12). EE phase results are presented here, and a new summary of safety findings across the entire study period is provided for a comprehensive assessment of levoketoconazole treatment. The median duration of exposure to levoketoconazole during EE was 5.8 (range, 0.4–8.9) months, resulting in a median levoketoconazole exposure for the entire study of 15 (range, 7.9–22) months in the EE population (*n*  = 60).

AEs were reported among 67% of patients during EE (Table 3). The most commonly reported AEs during EE were arthralgia, headache, hypokalemia, and QT prolongation (each in 4 (6.7%) patients). Most AEs (95%) were of mild or moderate intensity, but some severe AEs were reported: depression, hyperkalemia, hyperlipasemia, hypoglycemia, and QT prolongation (1 patient each). Serious AEs were reported in four patients (benign ovarian tumor, epilepsy, hyphema, hypoglycemia), none of which was considered to be related to levoketoconazole. Four (6.7%) patients discontinued during EE due to AEs (AST increased (*n* = 1), benign ovarian tumor (*n* = 1), palpitations (*n* = 1), QT prolongation (*n* = 2)). There were no reports of adrenal insufficiency or liver-related AEs of special interest during EE.

**Table 3:** Summary of treatment-emergent adverse events. Data are presented as *n* (%).

Adverse event	Extended evaluation phase	All phases combined
*n*	60	94
Any AE	40 (66.7)	92 (97.9)
Serious AE	4 (6.7)	16 (17.0)
Drug-related AE*	6 (10.0)	42 (44.7)
AE leading to discontinuation	4 (6.7)^†^	16 (17.0)
Intensity of AEs^‡^		
Mild	21 (35.0)	14 (14.9)
Moderate	14 (23.3)	57 (60.6)
Severe	5 (8.3)	19 (20.2)
Life-threatening	0 (0)	1 (1.1)
Most common AEs		
Incidence ≥ 5%^§^		
Arthralgia	4 (6.7)	
Headache	4 (6.7)	
Hypokalemia	4 (6.7)	
QT prolongation	4 (6.7)	
Nasopharyngitis	3 (5.0)	
Incidence ≥15%^⁋^		
Nausea		31 (33.0)
Headache		27 (28.7)
Hypertension		18 (19.1)
Peripheral edema		18 (19.1)
Fatigue		17 (18.1)
ALT increased		16 (17.0)
Diarrhea		15 (16.0)

^*^AEs that were assessed by the investigator as probably or definitely related to levoketoconazole; ^†^AST increased (*n*  = 1), benign ovarian tumor (*n*  = 1), palpitations (*n*  = 1), and QT prolongation (*n*  = 2); all except the tumor were considered treatment-related; ^‡^Each patient with ≥1 treatment-emergent AE was counted only once, under the worst intensity experienced; ^§^In the extended evaluation phase; ^⁋^In all phases combined.

AE, adverse event; ALT, alanine aminotransferase; AST, aspartate aminotransferase.

During EE, serum alanine aminotransferase (ALT) and aspartate aminotransferase (AST) concentrations between ULN and ≤3× ULN were observed in 4 (7.4%) and 5 (9.3%) of 54 patients, respectively, with no values >3× ULN. The four patients with ALT >ULN during EE also had ≥1 abnormal ALT value prior to EE, while three of the five patients with abnormal AST had ≥1 pre-EE phase elevated AST value. Across all study phases combined, 39 (41%) of 94 patients had a maximal value in one or both transaminases that exceeded the ULN at any time postbaseline, with 8.5% maximally between >3× ULN and ≤5× ULN and 3.2% maximally >5× ULN.

Four (6.7%) of 60 patients were reported with one or more AEs of QT prolongation during EE. Two (3.3%) of 60 patients had a QT prolongation AE of special interest, and 3 (5.6%) of 54 patients had at least 1 Fridericia-corrected QT (QTcF) interval longer than 450 ms; however, no QTcF intervals were longer than 460 ms during EE. Of the three patients with QTcF intervals exceeding 450 ms, one had a similarly elevated QTcF prior to EE. No patient had a change from baseline in QTcF exceeding 60 ms during EE. Across all study phases combined, 5 (5.3%) of 94 patients had an AE of QT prolongation, 9 (10.2%) of 88 patients had at least one QTcF interval representing an increase of more than 60 ms from baseline, and 25 (26.6%) and 2 (2.1%) of 94 patients had one or more confirmed QTcF intervals longer than 450 and 500 ms, respectively. No arrhythmias secondary to QT prolongation were reported.

In female patients, mean total, free, and bioavailable testosterone concentrations decreased significantly from baseline at months 6, 9, and 12 (Table 4). In male patients who were not receiving testosterone therapy (*n*  = 7), mean total testosterone increased significantly only at month 12.

**Table 4:** Testosterone concentrations in males and females (extended evaluation population).

Parameters	Study baseline		Month 6		*P* value*	Month 9		*P* value*	Month 12		*P* value*
	Mean ± s.d.	*n*	Mean ± s.d.	*n*		Mean ± s.d.	*n*		Mean ± s.d.	*n*	
Females											
Free testosterone, nmol/L	0.011 ± 0.011	47	0.004 ± 0.006	44	<0.0001	0.003 ± 0.005	44	<0.0001	0.003 ± 0.005	36	<0.001
Bioavailable testosterone, nmol/L	0.23 ± 0.20	47	0.10± 0.10	44	<0.0001	0.09 ± 0.08	44	<0.0001	0.09 ± 0.08	36	0.0002
Total testosterone, nmol/L	0.88 ± 0.55	47	0.52 ± 0.36	44	<0.0001	0.52 ± 0.36	44	<0.0001	0.48 ± 0.30	37	<0.001
Males^†^											
Free testosterone, nmol/L	0.15 ± 0.03	7	0.21 ± 0.17	6	0.4623	0.22 ± 0.20	7	0.4340	0.22 ± 0.06	6	0.1038
Bioavailable testosterone, nmol/L	3.0 ±0.6	7	4.5 ± 4.1	6	0.4305	4.3 ± 4.1	7	0.4277	4.1 ± 1.1	6	0.1442
Total testosterone, nmol/L	10.8 ± 5.0	7	20.5 ± 12.8	6	0.1108	18.0 ± 12.1	7	0.1483	17.9 ± 4.8	6	0.0244

*Two-sided *P* value from the paired *t*-test performed on the change from baseline to months 6, 9, and 12.

^†^Males on testosterone therapy (*n*  = 3) were excluded.

Levoketoconazole daily dose was down-titrated in only three patients from month 6 to month 9, and no patients had their daily dose down-titrated from month 9 to month 12. Daily dose was down-titrated in two patients because of AEs (vomiting in one patient; fatigue, pain in extremity, and neck pain in the other patient). Of the seven patients who were not adherent to treatment in the EE phase, six (86%) had at least one AE, with all AEs mild (50%) or moderate (50%) in intensity and none leading to treatment discontinuation.

## Discussion

The SONICS study demonstrated the efficacy of levoketoconazole in normalizing mUFC during dose-titration and durability of response via a fixed-dose, 6-month maintenance phase (primary endpoint), and also provided evidence of improvement in biomarkers of CS comorbidities at the end of the maintenance phase (12, 20, 21). The prospective extended evaluation of the SONICS study described here included safety and efficacy evaluations through a median total exposure of approximately 15 months and further supports a role for levoketoconazole in long-term medical therapy for patients with endogenous CS.

Achieving and maintaining normal UFC levels is a key treatment goal in CS, as are improvements in comorbidities and signs and symptoms of the syndrome that contribute to decrements in QoL (1, 7, 24). Levoketoconazole efficacy observed during the SONICS study maintenance phase, with respect to mUFC, CS comorbidity biomarkers, clinical signs and symptoms of CS, QoL, and symptoms of depression (12), was generally sustained in this 6-month EE phase. Influence on cortisol diurnal rhythm has not been demonstrated consistently, as mean change in LNSC level was significant only at month 6. The majority of patients were receiving relatively low doses of levoketoconazole (600 mg/day or less) during the EE phase; increasing the daily dose and/or dividing the dose unequally, with a greater proportion administered in the evening vs the morning, have been suggested as options for improving diurnal cortisol control with steroidogenesis inhibitors (25).

Importantly, concentration-response modeling showed that any decline in mUFC response rate at the final study visit could not be explained by response tolerance owing to auto-induction of drug metabolism, as this appears to be maximal within ~2 weeks after starting treatment or increasing the dose. This model, without a tolerance effect applied, fits the observed UFC data well through 6 months of maintenance (data on file; Xeris Biopharma). There is also no evidence of loss of efficacy over time (‘escape’) due to increased ACTH drive, potentially overcoming levoketoconazole-induced blockade of cortisol synthesis. As shown in the current analyses, there was no correlation between changes from baseline in plasma ACTH and mUFC levels. Furthermore, plasma ACTH concentrations were similar between patients with CD who had normal and above-normal mUFC at month 12.

CS is associated with cardiovascular disease and metabolic disturbances, which are the leading causes of death in this disorder (4). Therefore, it is important to highlight that levoketoconazole treatment produced significant mean reductions from baseline in fasting glucose, HbA1c, and total and LDL cholesterol in the EE phase, similar to reductions observed at month 6 (12). Consistent with clinician-rated improvements in acne and hirsutism, mean testosterone concentrations in women decreased significantly from baseline at month 6 and were maintained at a similar level in EE phase. In contrast, cortisol synthesis inhibitors with a different mechanism of action, such as osilodrostat and metyrapone, tend to have opposite effects to increase testosterone, and in some women worsen acne and hirsutism (26, 27, 28, 29). Maintenance of testosterone within the normal range among men who were not using testosterone replacement was an unexpected finding and should be interpreted cautiously because of the small sample size.

There are scant longitudinal data on the effect of adrenal steroidogenesis inhibitors on pituitary adenoma growth (1, 30, 31). Although enlargement of corticotroph adenomas may be a concern with the use of steroidogenesis inhibitors, it is difficult to determine whether any observed change in tumor size is related to loss of ACTH-cortisol feedback or reflects the natural history of progressive or recurrent disease (32). In the only other prospective study (LINC 3) of an adrenal steroidogenesis inhibitor with reliable central MRI reading, more patients had a decrease in tumor volume (38%) than had an increase (33%) with up to 48 weeks of treatment with osilodrostat, despite an approximate doubling of plasma ACTH from baseline (26). Data for ketoconazole are available only from retrospective studies, the largest of which reported a newly visible tumor on MRI in eight (5%) patients during a mean (s.d.) follow-up period of 25 (33.6) months (33), while an earlier study had reported surgical treatment for five patients due to a de novo visible tumor 12–30 months after initiation of ketoconazole (34). Thus, in contrast to ketoconazole data, and in agreement with data from LINC 3 (osilodrostat), the current report showed that under levoketoconazole therapy the subset of patients with CD but without visible pituitary tumor at baseline did not subsequently develop a visible tumor during up to 20 months of follow-up. About 90% of patients with visible tumor at baseline had <2 mm change in the largest tumor diameter. A direct inhibitory effect of levoketoconazole on pituitary tumor growth was shown experimentally, which could counteract the effect of negative feedback withdrawal from cortisol lowering (10). Nevertheless, periodic monitoring of tumor size and location during long-term therapy is prudent (1), as significant tumor enlargement has been described during several years of medical therapy with osilodrostat in some cases (35).

Levoketoconazole was well tolerated in the EE phase of the SONICS study. Overall, the incidence of treatment-emergent AEs was substantially lower in the EE phase than in the first two phases of the study, likely a function of common events first occurring early during treatment without subsequent worsening. Indeed, the most commonly reported AEs during the first 2 phases occurred relatively infrequently in the EE: nausea for only one (1.7%) patient, headache for four (6.7%), hypertension for two (3.3%), and peripheral edema for none.

Hepatotoxicity is a known risk with racemic ketoconazole; the US prescribing information includes a boxed warning (36), and the European summary of product characteristics states that treatment with ketoconazole should not be initiated in patients with liver enzymes >2× ULN (37). In the EE phase of the SONICS study, no patient experienced ALT or AST >3× ULN; additionally, no patient had a change from baseline in QTcF exceeding 60 ms, nor an absolute interval duration exceeding 460 ms. Thus, the potentially clinically important events relating to suspected liver toxicity or QT interval prolongation were confined to the first two phases of the SONICS study. However, as changes in liver tests and QTc interval are frequently asymptomatic, continued periodic monitoring during long-term treatment is advisable to mitigate these potential risks.

Overall, SONICS safety findings support the conclusion that levoketoconazole may be safely used long-term in CS with monitoring that is initially frequent and could be diminished in frequency once a stable therapeutic dose has been identified. The minimum safety monitoring interval of 3 months during the EE phase appeared appropriate to prevent treatment-related serious AEs (none reported) and limit treatment-related AEs of any severity that led to discontinuation (QT prolongation: 2 (3.3%) patients; AST increased: 1 (1.7%) patient, none of which required treatment or had clinical sequelae). Comparisons to other medical therapies should be interpreted with caution in the absence of head-to-head studies; however, it appears that testosterone levels may be lower in women treated with levoketoconazole vs osilodrostat or metyrapone (1, 8). Similar to ketoconazole, levoketoconazole is a substrate and potent inhibitor of CYP3A4 and has the potential for drug–drug interactions (8).

Key limitations of the SONICS study are the open-label design and absence of a control group, as well as non-volumetric assessment of pituitary tumor size. However, MRI images were centrally read by a specialized pituitary neurosurgeon expert in neuroradiology, which decreased reader variability. A limitation of the extended evaluation specifically is the exploratory intention of the efficacy analyses. All efficacy results from the EE phase should, therefore, be considered as hypothesis-generating rather than confirmatory. In addition, study attrition owing to AEs or lack of efficacy tends to enrich the remaining study population with patients who benefit from and tolerate treatment. Another potential limitation is that seven patients who had prior radiation therapy were included in the extended evaluation, because prior therapy radiation may have impacted long-term cortisol control in these patients.

Among unexplored issues is the direct comparison of levoketoconazole with ketoconazole, the racemate from which it is derived, as there are no clinical studies of any type comparing levoketoconazole to ketoconazole. While the safety and efficacy profile of levoketoconazole for use in CS has been established in prospective clinical trials (12), ketoconazole has not been similarly studied. In the United States, levoketoconazole is approved for use in patients with CS of any etiology for whom surgery is not an option or when not curative, and labeling provides guidance for patient selection, dosing, and monitoring to support safe and effective use (9). However, levoketoconazole is not registered in Europe. In contrast, ketoconazole is registered for the treatment of CS in Europe, but in the United States, it is frequently used off-label for the treatment of CS, although US labeling for ketoconazole cautions against its use in CS (36).

Restrictions on the use of levoketoconazole include active liver disease and predisposition to QT prolongation or torsades de pointes, and levoketoconazole labeling contains a box warning for liver abnormalities and QTc prolongation. Both levoketoconazole and ketoconazole require ongoing monitoring of dosing and safety markers to assure appropriate use (9, 36). Though approximately 30% of patients in the SONICS study did not have prior surgery (12), it remains to be determined whether levoketoconazole is useful in the presurgical setting, either to delay the need for or to improve surgery outcomes.

In conclusion, in the 6-month EE phase of the SONICS study, levoketoconazole was well tolerated, and no new drug-related safety signals were observed during up to 22 months of treatment. Pituitary adenoma growth occurred in a minority of patients with CD but did not impact treatment. Sustained mUFC responses and improvements in CS comorbidity biomarkers, clinical signs and symptoms of CS, and QoL were demonstrated in most patients who completed the EE phase. These findings support the role of levoketoconazole in the long-term management of patients with CS.

## Supplementary Material

Supplementary Table S1. Study Sites and Corresponding Independent Ethics Committees

Supplementary Table S2. Levoketoconazole dose at Month 6 (extended evaluation population)

Supplementary Table S3. Changes from study baseline in mUFC, LNSC, and random serum cortisol levels (extended evaluation population)

Supplementary Table S4. Changes from Month 6 in mUFC and LNSC (extended evaluation population)

Supplementary Table S5. mUFC concentrations in patients who had prior radiation therapy at Months 6, 9, and 12 (extended evaluation population)

Supplementary Table S6. ACTH levels in patients with Cushing’s disease based on mUFC normalization status at Month 12 (extended evaluation population)

## Declaration of interest

MF reports serving as an investigator with research grants to OHSU from Novartis/Recordati and Strongbridge Biopharma (Strongbridge Biopharma is a wholly owned subsidiary of Xeris Biopharma Holdings, Inc.); serving as an occasional scientific consultant to HRA Pharma, Recordati, Sparrow, and Strongbridge Biopharma; and being Deputy Editor of *European Journal of Endocrinology*. RJA reports receiving research grants to the University of Michigan from Corcept Therapeutics, Novartis, and Strongbridge Biopharma; and serving as a consultant to Corcept Therapeutics, Crinetics Pharmaceuticals, H Lundbeck A/S, Novartis, Recordati Rare Diseases, and Strongbridge Biopharma. YG reports serving as the principal investigator of research grants to Tel Aviv-Sourasky Medical Center from Chiasma, Novartis, Strongbridge Biopharma, and Corcept Therapeutics; and receiving lecture fees from Medison, Novartis, and Pfizer. SZ reports receiving consulting honoraria from Novartis. EBG reports serving as an investigator for research grants to Memorial Sloan Kettering Cancer Center from Corcept Therapeutics, Novartis/Recordati, and Strongbridge Biopharma; and serving as an occasional scientific consultant to HRA Pharma and Strongbridge Biopharma/Xeris Biopharma. RS reports receiving an educational grant to Johns Hopkins University from Corcept Therapeutics; serving as an investigator with research grants to Johns Hopkins University from Crinetics and Strongbridge Biopharma; and serving as a consultant for HRA Pharma Rare Disease, Ipsen, NovoNordisk, Recordati Rare Diseases, and Strongbridge Biopharma. RP reports serving as the principal investigator of research grants to Federico II University from Corcept Therapeutics, Novartis, Recordati, and Strongbridge Biopharma; and receiving consulting honoraria from Novartis, Recordati, and Strongbridge Biopharma. UFR reports receiving speaker honoraria from Novartis, Novo Nordisk, and Pfizer; and serving on advisory boards for Novartis, Novo Nordisk, Recordati, and Pfizer. UFR’s research salary was sponsored by a grant from Kirsten and Freddy Johansen’s Fund. LK reports serving as a principal investigator of research grants to the Cleveland Clinic from Chiasma, Crinetics, Ionis, and Strongbridge Biopharma; receiving consulting honoraria from Corcept Therapeutics and Novo Nordisk; and receiving speaker fees from Corcept Therapeutics. MB reports receiving research grants to the University of Erlangen-Nuremberg from Strongbridge Biopharma. BMKB reports serving as the principal investigator of research grants to Massachusetts General Hospital from Strongbridge Biopharma; and receiving consulting honoraria from HRA Pharma, Novartis/Recordati, Sparrow, and Strongbridge Biopharma/Xeris Biopharma. FC is a former employee of Strongbridge Biopharma/Xeris Biopharma and reports receiving consulting fees from Xeris Biopharma. APH reports serving as a principal investigator of research grants to UCLA from Chiasma, Crinetics, Ionis, Ascendis, and Strongbridge Biopharma; and receiving consulting honoraria from Ipsen, Novo Nordisk, Lundbeck, and Strongbridge Biopharma.

## Funding

This study was funded by Cortendo AB (a subsidiary of Strong http://dx.doi.org/10.13039/100014345bridge Biopharma (now Xeris Biopharma)).

## SONICS Investigator List

Principal investigators (*n* = number of patients enrolled in the study for each site): Belgium: Marie Bex (University Hospitals Leuven; *n* = 3); Bulgaria: Sabina Zacharieva (Acad. Ivan Penchev; *n* = 6); Canada: Ehud Ur (St. Pauls Hospital/Vancouver General Hospital; *n* = 1); Czechia: Vaclav Hana (Vseobecna fakultni nemocnic v Praze – III. Interni klinika VFN a 1. LF UK; *n* = 0); Denmark: Marianne Andersen (Odense Universitets Hospital; *n* = 0); Ulla Feldt-Rasmussen (Rigshospitalet, Copenhagen University Hospital; *n* = 3); Caroline Kistorp (Herlev Hospital, Research Unit; *n* = 0); Logstrup Poulsen (Aarhus University Hospital; *n* = 1); France: Thierry Brue (Hopital de la CONCEPTION Service d’Endocrinologie, Diabete et Maladies Metaboliques; *n* = 2); Georgia: David Metreveli (David Metreveli Medical Centre; *n* = 0); Germany: Georg Brabant (Med Clinic I - University of Lubeck; *n* = 1); Gunter Stalla (Max-Plack-Institute of Psychiatry; *n* = 0); Hungary: Laszlo Kovacs (MH – Egeszsegugyi Kozpont; *n* = 0); Miklos Toth (Semmelweis University; *n* = 0); Israel: Faiad Adawi (Ziv Medical Center; *n* = 0); Yona Greenman (Sourasky Medical Center; *n* = 4); Leonard Saiegh (Bnai Zion Medical Center Institute of Endocrinology and Metabolism; *n* = 3); Ilan Shimon (Institute of Endocrinology and Metabolism, Rabin Medical Center, Beilinson Campus; *n* = 2); Italy: Giorgio Arnaldi (Azienda Ospedaliera - Universitaria Ancona; *n* = 3); Salvatore Cannavo (UOC di Endocrinologia, Dipartimento di Medicina, AOU Policlinico G. Martino; *n* = 2); Maria Vittorai Davi (Policlinico GB Rossi; *n* = 0); Diego Ferone (University of Genova, IRCCS AOU San Martino-IST; *n* = 0); Roberta Giordano (Azienda Ospedaliero - Universitaria Città della Salute e della Scienza di Torino; *n* = 0); Massimo Mannelli (Azienda Ospedaliero - Universitaria Careggi; *n* = 0); Francesca Pecori Giraldi (Istituto Auxologico Italiano; *n* = 3); Rosario Pivonello (University of Naples Federico II; *n* = 6); Alfredo Pontecorvi (Policlinico Universitario Agostino Gemelli; *n* = 1); Carla Scaroni (University of Padua; *n* = 2); Massimo Terzolo (SCDU Medicina Interna I, Universita di Torino; *n* = 3); Vincenzo Toscano (UOC Endocrinologia, Azienda Ospedaliera Sant’Andrea; *n* = 0); Netherlands: Nienke Biermasz (Leiden University Medical Center; *n* = 1); Richard Feelders (Polikliniek Endocrinologie, Erasmus MC; *n* = 4); Poland: Marek Bolanowski (Samodzielny Publiczny Szpital Kliniczny Nr 1; *n* = 1); Andrzej Lewinski (Instytut Centrum Zdrowia Matki Polki; *n* = 1); Beata Matyjaszek-Matuszek (Terpa Sp.z.o.o; *n* = 2); Marek Ruchala (Szpital Kliniczny im. Heliodora Swiecickiego; *n* = 0); Przemyslaw Witek (Outpatient Clinic: Reuma Centrum; *n* = 4); Serbia: Milica Medic-Stojanoska (Clinical Center of Vojvodina Clinic for Endocrinology; *n* = 0); Sandra Pekic-Djurdjevic (Clinical Center of Serbia; *n* = 1); Spain: Carmen Fajardo (Hospital Universidad de la Ribera; *n* = 1); Maria Angeles Galvez (Hospital Universitario Reina Sofia;*n* = 1); Susan Webb (Hospital de la Santa Creu i Sant Pau; *n* = 1); Sweden: Gudmundur Johannsson (Sahlgrenska University Hospital; *n* = 0); Turkey: Abdurrahman Comlekci (Dokuz Eylul University Medical Faculty; *n* = 0); Pinar Kadioglu (Istanbul University Medical Faculty; *n* = 1); Ertugrul Tasan (Bezmi Alem Vakif Universitesi Endokrinoloji Bolumu Adnan; *n* = 2); UK: Tara Kearney (Salford Royal NHS Foundation Trust; *n* = 0); David Ray (Manchester Royal Infirmary; *n* = 0); USA: Richard Auchus (University of Michigan Medical Center; *n* = 4); Beverly Biller (Massachusetts General Hospital; *n* = 1); Maria Fleseriu (Oregon Health and Science University; *n* = 5); Eliza Geer (Memorial Sloan Kettering Cancer Center; *n* = 2; Icahn School of Medicine at Mount Sinai; *n* = 2); Hans Ghayee (University of Florida; *n* = 0); Murray Gordon (Allegheny Neuroendocrinology Center; *n* = 2); Anthony Heaney (University of California, Los Angeles, School of Medicine; *n* = 3); Patricia Kapsner (University of New Mexico HSC; *n* = 1); Laurence Kennedy (Cleveland Clinic; *n* = 3); Roberto Salvatori (Johns Hopkins University; *n* = 5); Kevin Yuen (Swedish Hospital; *n* = 0).

## References

[1] Fleseriu M Auchus R Bancos I Ben-Shlomo A Bertherat J Biermasz NR Boguszewski CL Bronstein MD Buchfelder M Carmichael JD***et al***. Consensus on diagnosis and management of Cushing's disease: a guideline update. Lancet. Diabetes and Endocrinology 2021 9 847–875. (10.1016/S2213-8587(2100235-7) 34687601 PMC8743006

[2] Lacroix A Feelders RA Stratakis CA Nieman LK. Cushing's syndrome. Lancet 2015 386 913–927. (10.1016/S0140-6736(1461375-1) 26004339

[3] Biller BMK Grossman AB Stewart PM Melmed S Bertagna X Bertherat J Buchfelder M Colao A Hermus AR Hofland LJ***et al***. Treatment of adrenocorticotropin-dependent Cushing's syndrome: a consensus statement. Journal of Clinical Endocrinology and Metabolism 2008 93 2454–2462. (10.1210/jc.2007-2734) 18413427 PMC3214276

[4] Pivonello R Isidori AM De Martino MC Newell-Price J Biller BMK Colao A. Complications of Cushing's syndrome: state of the art. Lancet. Diabetes and Endocrinology 2016 4 611–629. (10.1016/S2213-8587(1600086-3) 27177728

[5] Ragnarsson O Olsson DS Papakokkinou E Chantzichristos D Dahlqvist P Segerstedt E Olsson T Petersson M Berinder K Bensing S***et al***. Overall and disease-specific mortality in patients with Cushing's disease: a Swedish nationwide study. Journal of Clinical Endocrinology and Metabolism 2019 104 2375–2384. (10.1210/jc.2018-02524) 30715394

[6] McBride MM Crespo I Webb SM Valassi E. Quality of life in Cushing's syndrome. Best Practice and Research. Clinical Endocrinology and Metabolism 2021 35 101505. (10.1016/j.beem.2021.101505) 33707083

[7] Nieman LK Biller BMK Findling JW Murad MH Newell-Price J Savage MO Tabarin A & Endocrine Society. Treatment of Cushing's syndrome: an Endocrine Society Clinical Practice Guideline. Journal of Clinical Endocrinology and Metabolism 2015 100 2807–2831. (10.1210/jc.2015-1818) 26222757 PMC4525003

[8] Fleseriu M Auchus RJ Pivonello R Salvatori R Zacharieva S Biller BMK. Levoketoconazole: a novel treatment for endogenous Cushing's syndrome. Expert Review of Endocrinology and Metabolism 2021 16 159–174. (10.1080/17446651.2021.1945440) 34380370

[9] RECORLEV. (Levoketoconazole) Tablets, for Oral Use: Chicago, IL: Xeris Pharmaceuticals, Inc 2021.

[10] Creemers SG Feelders RA de Jong FH Franssen GJH de Rijke YB van Koetsveld PM Hofland LJ. Levoketoconazole, the 2S,4R enantiomer of ketoconazole, a new steroidogenesis inhibitor for Cushing’s syndrome treatment. Journal of Clinical Endocrinology and Metabolism 2021 106 e1618–e1630. (10.1210/clinem/dgaa989) 33399817

[11] Thieroff-Ekerdt R Lavin P Abou-Gharbia M France NPEds. Pharmacology of COR-003 (levoketoconazole), an investigational treatment for endogenous Cushing’s syndrome. 98th Annual Meeting and Expo of the Endocrine Society (ENDO); Boston, MA; 2016.

[12] Fleseriu M Pivonello R Elenkova A Salvatori R Auchus RJ Feelders RA Geer EB Greenman Y Witek P Cohen F***et al***. Efficacy and safety of levoketoconazole in the treatment of endogenous Cushing’s syndrome (SONICS): a phase 3, multicentre, open-label, single-arm trial. Lancet. Diabetes and Endocrinology 2019 7 855–865. (10.1016/S2213-8587(1930313-4) 31542384

[13] Pivonello R Zacharieva S Elenkova A Toth M Shimon I Stigliano A Badiu C Brue T Georgescu CE Tsagarakis S***et al***. Levoketoconazole in the treatment of patients with endogenous Cushing's syndrome: a double-blind, placebo-controlled, randomized withdrawal study (LOGICS) (published online ahead of print September 9, 2022). Pituitary 2022. (10.1007/s11102-022-01263-7) PMC9675660 36085339

[14] Doshi A Zaheer A Stiller MJ. A comparison of current acne grading systems and proposal of a novel system. International Journal of Dermatology 1997 36 416–418. (10.1046/j.1365-4362.1997.00099.x) 9248884

[15] Hatch R Rosenfield RL Kim MH Tredway D. Hirsutism: implications, etiology, and management. American Journal of Obstetrics and Gynecology 1981 140 815–830. (10.1016/0002-9378(8190746-8) 7258262

[16] Brodovicz KG McNaughton K Uemura N Meininger G Girman CJ Yale SH. Reliability and feasibility of methods to quantitatively assess peripheral edema. Clinical Medicine and Research 2009 7 21–31. (10.3121/cmr.2009.819) 19251582 PMC2705274

[17] Webb SM Badia X Barahona MJ Colao A Strasburger CJ Tabarin A van Aken MO Pivonello R Stalla G Lamberts SWJ***et al***. Evaluation of health-related quality of life in patients with Cushing's syndrome with a new questionnaire. European Journal of Endocrinology 2008 158 623–630. (10.1530/EJE-07-0762) 18426820

[18] Beck AT Steer RA Ball R Ranieri WF. Comparison of Beck Depression Inventories -IA and -II in psychiatric outpatients. Journal of Personality Assessment 1996 67 588–597. (10.1207/s15327752jpa6703_13) 8991972

[19] Beck AT Steer RA Brown GK Beck Depression Inventory®, 2nd ed (BDI®–II): San Antonio, TX: The Psychological Corporation 1996.

[20] Geer EB Salvatori R Elenkova A Fleseriu M Pivonello R Witek P Feelders RA Bex M Borresen SW Puglisi S***et al***. Levoketoconazole improves clinical signs and symptoms and patient-reported outcomes in patients with Cushing’s syndrome. Pituitary 2021 24 104–115. (10.1007/s11102-020-01103-6) 33216275 PMC7864823

[21] Pivonello R Elenkova A Fleseriu M Feelders RA Witek P Greenman Y Geer EB Perotti P Saiegh L Cohen F***et al***. Levoketoconazole in the treatment of patients with Cushing’s syndrome and diabetes mellitus: results from the SONICS phase 3 study. Frontiers in Endocrinology 2021 12 595894. (10.3389/fendo.2021.595894) PMC8059833 33897615

[22] Nelson LM Forsythe A McLeod L Pulgar S Maldonado M Coles T Zhang Y Webb SM Badia X. Psychometric evaluation of the Cushing's Quality-of-Life questionnaire. Patient 2013 6 113–124. (10.1007/s40271-013-0012-5) 23575965

[23] Button KS Kounali D Thomas L Wiles NJ Peters TJ Welton NJ Ades AE Lewis G. Minimal clinically important difference on the Beck Depression Inventory--II according to the patient's perspective. Psychological Medicine 2015 45 3269–3279. (10.1017/S0033291715001270) 26165748 PMC4611356

[24] Castinetti F Nieman LK Reincke M Newell-Price J. Approach to the patient treated with steroidogenesis inhibitors. Journal of Clinical Endocrinology and Metabolism 2021 106 2114–2123. (10.1210/clinem/dgab122) 33675650 PMC8427736

[25] Yoshida K Fukuoka H Odake Y Nakajima S Tachibana M Ito J Hosokawa Y Yamada T Miura H Suematsu N***et al***. Multiple salivary cortisol measurements are a useful tool to optimize metyrapone treatment in patients with Cushing's syndromes treatment: case presentations. Frontiers in Endocrinology (Lausanne) 2017 8 375. (10.3389/fendo.2017.00375) PMC5768610 29375480

[26] Pivonello R Fleseriu M Newell-Price J Bertagna X Findling J Shimatsu A Gu F Auchus R Leelawattana R Lee EJ***et al***. Efficacy and safety of osilodrostat in patients with Cushing's disease (LINC 3): a multicentre phase III study with a double-blind, randomised withdrawal phase. Lancet. Diabetes and Endocrinology 2020 8 748–761. (10.1016/S2213-8587(2030240-0) 32730798

[27] Fleseriu M Pivonello R Young J Hamrahian AH Molitch ME Shimizu C Tanaka T Shimatsu A White T Hilliard A***et al***. Osilodrostat, a potent oral 11β-hydroxylase inhibitor: 22-week, prospective, phase II study in Cushing's disease. Pituitary 2016 19 138–148. (10.1007/s11102-015-0692-z) 26542280 PMC4799251

[28] Daniel E Aylwin S Mustafa O Ball S Munir A Boelaert K Chortis V Cuthbertson DJ Daousi C Rajeev SP***et al***. Effectiveness of metyrapone in treating Cushing's syndrome: a retrospective multicenter study in 195 patients. Journal of Clinical Endocrinology and Metabolism 2015 100 4146–4154. (10.1210/jc.2015-2616) 26353009 PMC5393433

[29] Pivonello R Ferrigno R De Martino MC Simeoli C Di Paola N Pivonello C Barba L Negri M De Angelis C Colao A. Medical treatment of Cushing's disease: an overview of the current and recent clinical trials. Frontiers in Endocrinology (Lausanne) 2020 11 648. (10.3389/fendo.2020.00648) PMC7753248 33363514

[30] Fleseriu M Findling JW Koch CA Schlaffer SM Buchfelder M Gross C. Changes in plasma ACTH levels and corticotroph tumor size in patients with Cushing's disease during long-term treatment with the glucocorticoid receptor antagonist mifepristone. Journal of Clinical Endocrinology and Metabolism 2014 99 3718–3727. (10.1210/jc.2014-1843) 25013998 PMC4399272

[31] Baudry C Coste J Bou Khalil R Silvera S Guignat L Guibourdenche J Abbas H Legmann P Bertagna X Bertherat J. Efficiency and tolerance of mitotane in Cushing's disease in 76 patients from a single center. European Journal of Endocrinology 2012 167 473–481. (10.1530/EJE-12-0358) 22815335

[32] Varlamov EV Han AJ Fleseriu M. Updates in adrenal steroidogenesis inhibitors for Cushing's syndrome - a practical guide. Best Practice and Research. Clinical Endocrinology and Metabolism 2021 35 101490. (10.1016/j.beem.2021.101490) 33707082

[33] Castinetti F Guignat L Giraud P Muller M Kamenicky P Drui D Caron P Luca F Donadille B Vantyghem MC***et al***. Ketoconazole in Cushing's disease: is it worth a try? Journal of Clinical Endocrinology and Metabolism 2014 99 1623–1630. (10.1210/jc.2013-3628) 24471573

[34] Castinetti F Morange I Jaquet P Conte-Devolx B Brue T. Ketoconazole revisited: a preoperative or postoperative treatment in Cushing's disease. European Journal of Endocrinology 2008 158 91–99. (10.1530/EJE-07-0514) 18166822

[35] Fontaine-Sylvestre C Létourneau-Guillon L Moumdjian RA Berthelet F Lacroix A. Corticotroph tumor progression during long-term therapy with osilodrostat in a patient with persistent Cushing's disease. Pituitary 2021 24 207–215. (10.1007/s11102-020-01097-1) 33074401

[36] Ketoconazole Tablets USP, 200 mg. Haifa Bay, Israel: Taro Pharmaceutical Industries Ltd. 2017.

[37] European Medicines Agency. Ketoconazole HRA 200 mg Tablets: Summary of Product Characteristics: London 2015.

