# Supplementary material for: Levoketoconazole treatment in endogenous Cushing’s syndrome: extended evaluation of clinical, biochemical, and radiologic outcomes
Source: Eur J Endocrinol. 2022 Oct 17;187(6):859–71. doi: 10.1530/EJE-22-0506 (PMC9716395; doi:10.1530/EJE-22-0506)
Supplement: Supplementary Table S1. Study Sites and Corresponding Independent Ethics Committees [file supplementary_table_1.pdf]

Supplementary Table S1. Study Sites and Corresponding Independent Ethics Committees

Country	Site Number	PI	Role	Site Name	CEC	LEC
**Belgium**	3201	Bex, Marie	National Coordinating Investigator	University Hospitals Leuven	Commissie Medische Ethiek<br>UZ KU Leuven / Onderzoek<br>U.Z. Gasthuisberg<br>Herestraat 49<br>B 3000 Leuven<br>Belgium	N/A
**Belgium**	3202	Velkeniers	Principal Investigator	Universitair Ziekenhuis Brussel	Commissie Medische Ethiek<br>UZ KU Leuven / Onderzoek<br>U.Z. Gasthuisberg<br>Herestraat 49<br>B 3000 Leuven<br>Belgium	Vrije Universiteit Brussel<br>Commissie Medische Ethiek<br>Reflectiegroep Biomedische Ethiek<br>Laarbeeklaan 101<br>1090 Brussel<br>Belgium
**Bulgaria**	3501	Zaharieva, Sabina	Principal Investigator	University Specialized Hospital for Active Treatment in Endocrinology (USHATE)<br>"Acad. Ivan Penchev"	Ethics Committee for Multicenter Clinical Trials<br>Ministry of Health<br>5 Sveta Nedelia Sq<br>Sofia, Bulgaria 1000	N/A
**Canada**	2003	Chik, Constance	Principal Investigator	University of Alberta	N/A	Health Research Ethics Board<br>308 Campus Tower<br>University of Alberta<br>Edmonton, Alberta<br>T6G 1K8, Canada
**Canada**	2001	Lacroix, Andre	Principal Investigator	Centre hospitalier de l'Université de Montréal (CHUM)	N/A	Comité d'éthique de la recherche de CHUM<br>Pavillon R, 900 rue St.-Denis, 3e étage<br>Montréal, QC<br>H2X 0A9, Canada<br>CHUM
**Canada**	2002	Ur, Ehud	Principal Investigator	St. Pauls Hospital/Vancouver General Hospital	N/A	The University of British Columbia<br>Office of Research (Services) Ethics<br>Clinical Research Ethics Board – Room 210, 828 West 10th Avenue,<br>Vancouver, BC<br>V5Z 1L8, Canada
**Czech Republic**	4201	Cap, Jan	National Coordinating Investigator	University Hospital Hradec Kralove	Etická komise<br>Fakultní nemocnice Hradec Králové<br>Sokolská 581<br>500 05 Hradec Králové<br>Czech Republic	N/A
**Czech Republic**	4202	Hana, Vaclav	Principal Investigator	Vseobecna fakultni nemocnice v Praze – III. Interni klinika VFN a 1. LF UK	Etická komise<br>Fakultní nemocnice Hradec Králové<br>Sokolská 581<br>500 05 Hradec Králové<br>Czech Republic	Eticka komise VFN<br>Na Bojisti 1<br>Prague, Czech Republic 128 08
**Czech Republic**	4203	Krsek, Michal	Principal Investigator	Fakultní nemocnice Královské Vinohrady, Prague	Etická komise<br>Fakultní nemocnice Hradec Králové<br>Sokolská 581<br>500 05 Hradec Králové<br>Czech Republic	Etická komise Fakultní nemocnice Královské Vinohrady<br>Šrobárova 1150/50, 100 34 Praha 10<br>Czech Republic

Country	Site Number	PI	Role	Site Name	CEC	LEC
**Denmark**	4505	Andersen, Marianne	Principal Investigator	Odense Universitets Hospital	Committees on Biomedical Research Ethics of the Capital Region<br>Regionsgården<br>Kongens Vænge 2<br>Hillerod, Denmark 3400	N/A
**Denmark**	4501	Feldt-Rasmussen, Ulla	National Coordinating Investigator	Rigshospitalet, Copenhagen University Hospital	Center for Health, the Committees on Health Research Ethics<br>Kongens Vaenge 2<br>Hillerod, Denmark 3400	N/A
**Denmark**	4504	Kistorp, Caroline	Principal Investigator	Herlev Hospital	Center for Health, the Committees on Health Research Ethics<br>Kongens Vaenge 2<br>Hillerod, Denmark 3400	N/A
**Denmark**	4502	Poulsen, Løgstrup	Principal Investigator	Aarhus University Hospital	Committees on Biomedical Research Ethics of the Capital Region<br>Regionsgården<br>Kongens Vænge 2<br>Hillerod, Denmark 3400	N/A
**France**	3304	Brue, Thierry	National Coordinating Investigator	Hôpital de la CONCEPTION<br>Service d'Endocrinologie, Diabète et Maladies Métaboliques	Center for Health, Region Hovedstaden, Ethics Committee<br>Kongens Vaenge 2<br>Hillerod, Denmark 3400	NA
**France**	3302	Chabre, Olivier	Principal Investigator	CHU de Grenoble, Hôpital Nord,<br>Service d'Endocrinologie-Diabétologie-Nutrition	Comité de Protection des Personnes Ile de France VIII<br>Hôpital Ambroise Paré<br>Bâtiment d'Anatomopathologie<br>9, avenue Charles de Gaulle<br>92100 Boulogne-Billancourt<br>France	NA
**France**	3307	Delemer, Brigitte	Principal Investigator	Hôpital Robert-Debré	Comité de Protection des Personnes Ile de France VIII<br>Hôpital Ambroise Paré<br>Bâtiment d'Anatomopathologie<br>9, avenue Charles de Gaulle<br>92100 Boulogne-Billancourt<br>France	N/A
**France**	3306	Goichot, Bernard	Principal Investigator	Hôpital de Hautepierre	Comité de Protection des Personnes Ile de France VIII<br>Hôpital Ambroise Paré<br>Bâtiment d'Anatomopathologie<br>9, avenue Charles de Gaulle<br>92100 Boulogne-Billancourt<br>France	N/A
**France**	3303	Tabarin, Antoine	Principal Investigator	CHU de Bordeaux, USN Haut-Levêque	Comité de Protection des Personnes Ile de France VIII<br>Hôpital Ambroise Paré<br>Bâtiment d'Anatomopathologie<br>9, avenue Charles de Gaulle<br>92100 Boulogne-Billancourt<br>France	N/A

Country	Site Number	PI	Role	Site Name	CEC	LEC
**France**	3305	Touraine, Philippe	Principal Investigator	Hôpitaux Universitaires Pitié Salpêtrière Charles Foix	Comité de Protection des Personnes Ile de France VIII<br>Hôpital Ambroise Paré<br>Bâtiment d'Anatomopathologie<br>9, avenue Charles de Gaulle<br>92100 Boulogne-Billancourt<br>France	N/A
**Germany**	4905	Brabant, Georg	Principal Investigator	Med Clinic I - University of Lueback	Ethikkommission bei der Medizinischen<br>Fakultät der Universität Würzburg<br>Institut für Pharmakologie und Toxikologie<br>Versbacher Str. 9<br>D-97078 Würzburg, Germany	Ethikkommission der Universität zu Lübeck<br>Ratzeburger Allee 160<br>D-23538 Lübeck, Germany
**Germany**	4901	Hahner, Stefanie	National Coordinating Investigator	University of Hospital Wuerzburg	Ethikkommission bei der Medizinischen<br>Fakultät der Universität Würzburg<br>Institut für Pharmakologie und Toxikologie<br>Versbacher Str. 9<br>D-97078 Würzburg, Germany	N/A
**Germany**	4903	Schopohl, Jochen	Principal Investigator	Klinikum der LMU	Ethikkommission bei der Medizinischen<br>Fakultät der Universität Würzburg<br>Institut für Pharmakologie und Toxikologie<br>Versbacher Str. 9<br>D-97078 Würzburg, Germany	Ethics Committee<br>Medical Faculty of the LMU München<br>Pettenkoferstr. 8a<br>80336 München, Germany
**Germany**	4902	Stalla, Günter	Principal Investigator	Max-Planck-Institute of Psychatry	Ethikkommission bei der Medizinischen<br>Fakultät der Universität Würzburg<br>Institut für Pharmakologie und Toxikologie<br>Versbacher Str. 9<br>D-97078 Würzburg, Germany	Ethics Committee<br>Medical Faculty of the LMU München<br>Pettenkoferstr. 8a<br>80336 München, Germany
**Germany**	4904	Strasburger, Christian	Principal Investigator	Charité University Medicine Berlin	Ethikkommission bei der Medizinischen<br>Fakultät der Universität Würzburg<br>Institut für Pharmakologie und Toxikologie<br>Versbacher Str. 9<br>D-97078 Würzburg, Germany	Charité - Universitätsmedizin Berlin<br>CCM: Campus Charité Mitte<br>Ethics Commission<br>Committee's Office<br>Charitéplatz 1<br>10117 Berlin, Germany
**Georgia**	5001	Metreveli, David	Principal Investigator	David Metreveli Medical Clinic	N/A	Ethics Committee<br>Tbilisi 0144 Tsinandali St. # 9;<br>Georgia
**Hungary**	3601	Kovács, László	National Coordinating Investigator	MH- Egészségügyi Központ	Medical Research Council Ethic Committee for Clinical Pharmacology (ETT_KFEB)<br>Arany Janos u. 6-8<br>Budapest, Hungary H-1051	N/A
**Hungary**	3602	Toth, Miklós	Principal Investigator	Semmelweis University	Medical Research Council Ethics Committee for Clinical Pharmacology (ECCP)<br>Arany Janos utca 6-8<br>Budapest, Hungary H-1051	N/A
**Israel**	9724	Adawi, Faiad	Principal Investigator	Ziv Medical Center	N/A	Ziv Medical Center Ethics Committee, Derech Rambam, Safed, Israel 13100
**Israel**	9722	Greenman, Yona	Principal Investigator	Sourasky Medical Center	N/A	Helsinki Committee, Tel Aviv Sourasky Medical Centre,<br>6 Weizman Street, Tel Aviv, Israel 64239
**Israel**	9723	Saiegh, Leonard	Principal Investigator	Bnai Zion Medical Center<br>Institute of Endocrinology & Metabolism	N/A	Helsinki Committee of Bnai Zion MC,<br>47 Golomb, Haifa, Israel 3104802
**Country**	**Site Number**	**PI**	**Role**	**Site Name**	**CEC**	**LEC**

**Israel**	9721	Shimon, Ilan	Principal Investigator	Institute of Endocrinology & Metabolism<br>Rabin Medical Center, Beilinson Campus	N/A	Rabin Medical Center<br>39 Jabotinski Street<br>Petach Tiqva, Israel 49100
**Italy**	3901	Arnaldi, Giorgio	National Coordinating Investigator	Azienda Ospedaliera-Universitaria Ancona	Comitato Etico Regionale delle Marche<br>Azienda Ospedaliero Universitaria Ancona<br>via Conca 71<br>Ancona, Italy 60126	N/A
**Italy**	3907	Cannavò, Salvatore	Principal Investigator	UOC di Endocrinologia<br>Dipartimento di Medicina Clinica e Sperimentale<br>Università degli Studi di Messina	Comitato Etico di Messina<br>AOU Policlinico G. Martino<br>via Consolare Valeria 1<br>Messina, Italy 98125	Spettabile<br>Comitato Etico<br>Policlinico G. Martino<br>Via Consolare Valeria 1<br>Messina, Italy 98125
**Italy**	3908	Ferone, Diego	Principal Investigator	University of Genova	Comitato Etico Regionale<br>Largo Rosanna Benzi, 10<br>Genoa, Liguria, Italy 16132	Spettabile<br>Comitato Etico<br>IRCCS AOU San Martino<br>Largo R. Benzi 10<br>Genova, Italy 16132
**Italy**	3906	Giordano, Roberta	Principal Investigator	Azienda Ospedaliero – Universitaria<br>Città della Salute e della Scienza di Torino	Azienda Ospedaliero Universitaria Ospedali Riuniti Umberto I- G.M. Lancisi- G. Salesi<br>via Conca 71<br>Ancona, Italy 60126	Spettabile<br>Comitato Etico<br>Città della Salute e della Scienza<br>padiglione beige, 3° piano (ex Casa Suore)<br>Corso Bramante, 88/90<br>Torino, Italy 10126
**Italy**	3910	Loli, Paola	Principal Investigator	ASST Grande Ospedale Metropolitano Niguarda	Azienda Ospedaliero Universitaria Ospedali Riuniti Umberto I- G.M. Lancisi- G. Salesi<br>via Conca 71<br>Ancona, Italy 60126	Spettabile<br>Comitato Etico<br>ASST Grande Ospedale Metropolitano Niguarda<br>Padiglione 7 – 4° Piano<br>Piazza Ospedale Maggiore 3<br>Milano, Italy 20162
**Italy**	3909	Davi, Maria	Principal Investigator	Policlinico GB Rossi	Azienda Ospedaliero Universitaria Ospedali Riuniti Umberto I- G.M. Lancisi- G. Salesi<br>via Conca 71<br>Ancona, Italy 60126	Spettabile Comitato Etico c/o Servizio di Farmacia dell'Ospedale Borgo Trento P.le Stefani 1,<br>Verona, Italy 37126
**Italy**	3913	Mannelli, Massimo	Principal Investigator	Mario Serio University of Florence	Azienda Ospedaliero Universitaria Ospedali Riuniti Umberto I- G.M. Lancisi- G. Salesi<br>via Conca 71<br>Ancona, Italy 60126	Comitato Etico Area Vasta Centro, Universitaria Careggi, Largo Brambilla 3,<br>Firenze, Italy 50134
**Italy**	3905	Pecori Giraldi, Francesca	Principal Investigator	Istituto Auxologico Italiano	Azienda Ospedaliero Universitaria Ospedali Riuniti Umberto I- G.M. Lancisi- G. Salesi<br>via Conca 71<br>Ancona, Italy 60126	Comitato Etico, Istituto Auxologico Italiano,<br>via L. Ariosto, 13,<br>Milan, Italy 20145
**Italy**	3903	Pivonello, Rosario	Principal Investigator	University of Naples Federico II	Azienda Ospedaliero Universitaria Ospedali Riuniti Umberto I- G.M. Lancisi- G. Salesi<br>via Conca 71<br>Ancona, Italy 60126	Comitato Etico Per le Attivita Biomediche Universita "Federico II", Via Sergio Pansini, 5,<br>Naples, Italy 80131
**Italy**	3911	Pontecorvi, Alfredo	Principal Investigator	Institute of Medical Pathology	Azienda Ospedaliero Universitaria Ospedali Riuniti Umberto I- G.M. Lancisi- G. Salesi<br>via Conca 71<br>Ancona, Italy 60126	Comitato Etico Policlinico Agostino Gemelli Universita Cattolica del Sacro Cuore, Largo Agostino Gemelli 8, Roma, Italy 00168

Country	Site Number	PI	Role	Site Name	CEC	LEC
**Italy**	3904	Scaroni, Carla	Principal Investigator	University of Padua	Azienda Ospedaliero Universitaria Ospedali Riuniti Umberto I- G.M. Lancisi- G. Salesi<br>via Conca 71<br>Ancona, Italy 60126	Comitato Etico per la Sperimentazione Clinica della Provincia di Padova, Via Giustiniani 1,<br>Padova, Italy 35128
**Italy**	3902	Terzolo, Massimo	Principal Investigator	SCDU Medicina Interna I Università di Torino	Azienda Ospedaliero Universitaria Ospedali Riuniti Umberto I- G.M. Lancisi- G. Salesi<br>Via Conca n.71<br>Ancona, Italy 60126	Spettabile<br>Comitato Etico<br>A.O.U. S. Luigi Gonzaga<br>Regione Gonzole 10<br>10043 Orbassano (TO)<br>Italy
**Italy**	3912	Toscano, Vincenzo	Principal Investigator	UOC Endocrinologia	Azienda Ospedaliero Universitaria Ospedali Riuniti Umberto I- G.M. Lancisi- G. Salesi<br>Via Conca n.71<br>Ancona, Italy 60126	Spettabile<br>Ufficio locale Sperimentazioni Cliniche<br>Comitato Etico dell'Università 'Sapienza'<br>c/o Azienda Ospedaliera Sant'Andrea<br>Via di Grottarossa 1035-1039<br>Roma, Italy 00189
**Netherlands**	3102	Biermasz, Nienke Ruurdje	Principal Investigator	Leiden University, Leiden University Medical Center	Medisch Ethische Toetsings Commissie van het ErasmusMC<br>'s Gravendijkwal 230<br>Rotterdam, Netherlands 3015 CE	N/A
**Netherlands**	3101	Feelders, Richard	National Coordinating Investigator	Erasmus MC	Medisch Ethische Toetsings Commissie van het ErasmusMC<br>'s Gravendijkwal 230<br>Rotterdam, Netherlands 3015 CE	N/A
**Poland**	4801	Bolanowski, Marek	Principal Investigator	Samodzielny Publiczny Szpital Kliniczny Nr 1	Bioethics Committee at the Polish Mother's Memorial Hospital Research Institute Rzgowska 281/289<br>Lodz, Poland 93338	N/A
**Poland**	4803	Lewinski, Andrzej	National Coordinating Investigator	Instytut Centrum Zdrowia Matki Polki	Bioethics Committee at the Polish Mother's Memorial Hospital Research Institute Rzgowska 281/289<br>Lodz, Poland 93338	N/A
**Poland**	4802	Matyjaszek-Matuszek, Beata	Principal Investigator	Terpa Sp.z.o.o	Bioethics Committee at the Polish Mother's Memorial Hospital Research Institute Rzgowska 281/289<br>Lodz, Poland 93338	N/A
**Poland**	4805	Ruchala, Marek	Principal Investigator	Szpital Kliniczny im. Heliodora Swiecickiego Uniwersytetu Medycznego im. Karola Marcinkowskiego w Poznaniu	Bioethics Committee at the Polish Mother's Memorial Hospital Research Institute Rzgowska 281/289<br>Lodz, Poland 93338	N/A
**Poland**	4804	Witek, Przemyslaw	Principal Investigator	Outpatient Clinic:<br>Reuma Centrum	Bioethics Committee at the Polish Mother's Memorial Hospital Research Institute Rzgowska 281/289<br>Lodz, Poland 93338	N/A
**Serbia**	3802	Medic-Stojanoska, Milica	Principal Investigator	Clinical Center of Vojvodina<br>Clinic for Endocrinology, Diabetes and Metabolic Diseases	N/A	Local Ethics Committee of the Clinical Center of Vojvodina<br>Clinical Center of Vojvodina<br>Hajduk Veljkova 1-11<br>Novi Sad, Serbia 21000
**Serbia**	3801	Pekic-Djurdjevic, Sandra	Principal Investigator	Clinical Center of Serbia	N/A	Local Ethics Committee of the Clinical Center of Serbia<br>Clinical Center of Serbia<br>Pasterova 2<br>Belgrade, Serbia 11000

Country	Site Number	PI	Role	Site Name	CEC	LEC
**Spain**	3403	Casanueva, Felipe	Principal Investigator	Hospital Provincial de Conxo	Ethics Committee of Clinical Research of Fundació de Gestió Sanitària del Hospital de la Santa Creu I Sant Pau<br>Servicio de Farmacologia Clinica Pabellon 19<br>Av. Sant Antoni M. Claret, 167<br>Barcelona, Spain 08025	N/A
**Spain**	3401	Montanana, Carmen Fajardo	Principal Investigator	Hospital Universidad De La Ribera	Comite Etico<br>Hospital Universitario de la Ribera<br>Carretera de Corbera KM 1<br>Alzira, Valencia, Spain 45600<br><br>Hospital de la Santa Creu i Sant Pau<br>Servicio de Farmacologia Clinica<br>Pabellon 19<br>Av. Sant Antoni M. Claret, 167<br>Barcelona, Spain 08025	N/A
**Spain**	3406	Galvez Moreno, Maria Angeles	Principal Investigator	Hospital Universitario Reina Sofía	Ethics Committee of Clinical Research of Fundació de Gestió Sanitària del Hospital de la Santa Creu I Sant Pau<br>Servicio de Farmacologia Clinica Pabellon 19<br>Av. Sant Antoni M. Claret, 167<br>Barcelona, Spain 08025	N/A
**Spain**	3402	Webb, Susan	National Coordinating Investigator	Hospital de la Santa Creu i Sant Pau	CEIC Hospital de la Santa Creu i Sant Pau<br>Sant Antoni Maria Claret,<br>167 Barcelona, Spain 08025	N/A
**Sweden**	4603	Engström, Britt Edén	Principal Investigator	University Hospital	Regional Ethis Committee in Gothenburg<br>Box 401 Gothenburg 405 30 Sweden	N/A
**Sweden**	4602	Höybye, Charlotte	Principal Investigator	Karolinska University Hospital	Regional Ethis Committee in Gothenburg<br>Box 401 Gothenburg 405 30 Sweden	N/A
**Sweden**	4601	Johannsson, Gudmundur	National Coordinating Investigator	Sahlgrenska University Hospital	Regional Ethis Committee in Gothenburg<br>Box 401 Gothenburg 405 30 Sweden	N/A
**Switzerland**	4101	Johannsson, Gudmundur	Principal Investigator	University Hospital Basel	EKNZ<br>Hebelstrasse 53<br>Basel 4056 Switzerland	N/A
**Turkey**	9001	Comlekci, Abdurrahman	Principal Investigator	Dokuz Eylul University Medical Faculty	Dokuz Eylul University Ethics Committee<br>Dokuz Eyul Universitesi Tip Fakultesi Yeni Dekanlik Binasi<br>Izmir 35340 Turkey	N/A
**Turkey**	9002	Kadioglu, Pinar	Principal Investigator	Istanbul University Medical Faculty	Bezmialem Vakif University Clinical Research Ethics Committee<br>Vatan Cad. Adnan Menderes Bulvari<br>Istanbul 34093 Turkey	N/A
**Turkey**	9003	Tasan, Ertugrul	Principal Investigator	Bezmi Alem Vakıf Üniversitesi Endokrinoloji Bölümü Adnan	Dokuz Eylul University Ethics Committee<br>Dokuz Eyul Universitesi Tip Fakultesi Yeni Dekanlik Binasi<br>Izmir 35340 Turkey	N/A
**UK**	4403	Aylwin, Simon	National Coordinating Investigator	Kings College Hospital	NRES Committee London-City & East<br>Bristol Research Ethics Committee Centre,<br>Level 3, Block B<br>Whitefriars, Lewins Mead, Level 3, Block B<br>Bristol, UK BS1 2NT	N/A

Country	Site Number	PI	Role	Site Name	CEC	LEC
**UK**	4406	Drake, William	Principal Investigator	St Bartholomew's Hospital	NRES Committee London-City & East<br>Bristol Research Ethics Committee Centre,<br>Level 3, Block B<br>Whitefriars, Lewins Mead, Level 3, Block B<br>Bristol, UK BS1 2NT	N/A
**UK**	4404	Kearney, Tara	Principal Investigator	Salford Royal NHS Foundation Trust	Research and Development<br>Department, Salford Royal NHS<br>Foundation Trust<br>Stott Lane<br>Salford, UK M68HD<br><br>NRES Committee London-City & East<br>Bristol Research Ethics Committee Centre,<br>Level 3, Block B<br>Whitefriars, Lewins Mead, Level 3, Block B<br>Bristol, UK BS1 2NT	N/A
**UK**	4405	Ray, David	Principal Investigator	Manchester Royal Infirmary	NRES Committee London-City & East<br>Bristol Research Ethics Committee Centre,<br>Level 3, Block B<br>Whitefriars, Lewins Mead, Level 3, Block B<br>Bristol, UK BS1 2NT	N/A
**UK**	4401	Trainer, Peter	Principal Investigator	Christie Hospital	NRES Committee London-City & East<br>Bristol Research Ethics Committee Centre,<br>Level 3, Block B<br>Whitefriars, Lewins Mead, Level 3, Block B<br>Bristol, UK BS1 2NT	N/A
**USA**	1024	Al-Karadsheh, Amer	Principal Investigator	Endocrine Center of Houston	Western Institutional Review Board<br>1019 39th Avenue SE, Suite 120<br>Puyallup, WA 98374-2115 USA	
**USA**	1001	Auchus, Richard	Principal Investigator	University of Michigan Medical Center		IRBMED<br>2800 Plymouth Road<br>Ann Arbor, MI 48109 USA
**USA**	1004	Biller, Beverly	Principal Investigator	Massachusetts General Hospital		Partners Human Research Committee<br>116 Huntington Avenue<br>Boston, MA 02116 USA
**USA**	1016	Chochinov, Ronald	Principal Investigator	Coastal Metabolic Research Center	Western Institutional Review Board<br>1019 39th Avenue SE, Suite 120<br>Puyallup, WA 98374-2115 USA	
**USA**	1019	De Santis, Pascual	Principal Investigator	Cleveland Clinic Florida		Cleveland Clinic Foundation - Institutional Review Board<br>9500 Euclid Avenue<br>Cleveland, OH 44195 USA
**USA**	1006	Fleseriu, Maria	Principal Investigator	Oregon Health & Science University		OHSU Research Integrity<br>3181 SW Sam Jackson Park Rd<br>Mail Code L106-R1<br>Portland, OR 97239 USA
**USA**	1011	Geer, Eliza	Principal Investigator	Icahn School of Medicine at Mount Sinai		Program for the Protection of Human Subjects, Mount Sinai<br>School of Medicine<br>One Gustave L. Levy Place<br>Box 1081<br>New York, NY 10029 USA

Country	Site Number	PI	Role	Site Name	CEC	LEC
**USA**	1026	Geer, Eliza	Principal Investigator	Memorial Sloan Kettering Cancer Center		Memorial Sloan Kettering Cancer Center Institutional Review Board/Privacy Board (IRB/PB)<br>1275 York Avenue<br>New York, NY 10065 USA
**USA**	1018	Ghayee, Hans	Principal Investigator	University of Florida	Western Institutional Review Board<br>1019 39th Avenue SE, Suite 120<br>Puyallup, WA 98374-2115 USA	
**USA**	1007	Gopalakrishnan, Geetha	Principal Investigator	Rhode Island Hospital, Hallett Center for Diabetes and Endocrinology		Lifespan Investigational Review Board<br>Coro West, Suite 1.3<br>One Hoppin Street<br>Providence, RI 02914 USA
**USA**	1021	Gordon, Murray	Principal Investigator	Allegheny Neuroendocrinology Center	Western Institutional Review Board<br>1019 39th Avenue SE, Suite 120<br>Puyallup, WA 98374-2115 USA	
**USA**	1014	Heaney, Anthony	Principal Investigator	UCLA School of Medicine		UCLA Office of the Human Research Protection Program<br>11000 Kinross Avenue<br>Los Angeles, CA 90095 USA
**USA**	1012	Iweha, Chioma	Principal Investigator	Advanced Research	Western Institutional Review Board<br>1019 39th Avenue SE, Suite 120<br>Puyallup, WA 98374-2115 USA	
**USA**	1008	Kapsner, Patricia	Principal Investigator	University of New Mexico HSC	Western Institutional Review Board<br>1019 39th Avenue SE, Suite 120<br>Puyallup, WA 98374-2115 USA	
**USA**	1003	Kennedy, Laurence	Principal Investigator	Cleveland Clinic		Cleveland Clinic Foundation - Institutional Review Board<br>9500 Euclid Avenue/OS-1<br>Cleveland, OH 44195 USA
**USA**	1009	Kirschner, Lawrence	Principal Investigator	Ohio State University Wexner Medical Center	Western Institutional Review Board<br>1019 39th Avenue SE, Suite 120<br>Puyallup, WA 98374-2115 USA	
**USA**	1023	Perley, Michael	Principal Investigator	Advanced Research	Western Institutional Review Board<br>1019 39th Avenue SE, Suite 120<br>Puyallup, WA 98374-2115 USA	

Country	Site Number	PI	Roles	Site Name	CEC	LEC
**USA**	1010	Salvatori, Roberto	Principal Investigator	Johns Hopkins University		Johns Hopkins Institutional Review Boards<br>Reed Hall - B130<br>1620 McElderry Street<br>Baltimore, MD 21205-1911 USA
**USA**	1027	Sheikh-Ali, Mae	Principal Investigator	East Coast Inst. For Research		IRB
**USA**	1005	Snyder, Peter	Principal Investigator	University of Pennsylvania		IRB
**USA**	1015	Yuen, Kevin	Principal Investigator	Swedish Hospital	Western Institutional Review Board<br>1019 39th Avenue SE, Suite 120<br>Puyallup, WA 98374-2115 USA	
