# Supplementary material for: Levoketoconazole treatment in endogenous Cushing’s syndrome: extended evaluation of clinical, biochemical, and radiologic outcomes
Source: Eur J Endocrinol. 2022 Oct 17;187(6):859–71. doi: 10.1530/EJE-22-0506 (PMC9716395; doi:10.1530/EJE-22-0506)
Supplement: Supplementary Table S2. Levoketoconazole dose at Month 6 (extended evaluation population) [file supplementary_table_2.pdf]

Supplementary Table S2. Levoketoconazole dose at Month 6 (extended evaluation population)

Patients by dose, *n* (%)	**Levoketoconazole dose at Month 6 of the Maintenance Phase (mg/day)**								**Overall**
	150	300	450	600	750	900	1050	1200	
	1 (1.7)	15 (25.0)	12 (20.0)	11 (18.3)	7 (11.7)	4 (6.7)	5 (8.3)	5 (8.3)	60
