# Supplementary material for: Levoketoconazole treatment in endogenous Cushing’s syndrome: extended evaluation of clinical, biochemical, and radiologic outcomes
Source: Eur J Endocrinol. 2022 Oct 17;187(6):859–71. doi: 10.1530/EJE-22-0506 (PMC9716395; doi:10.1530/EJE-22-0506)
Supplement: Supplementary Table S3. Changes from study baseline in mUFC, LNSC, and random serum cortisol levels (extended evaluation population) [file supplementary_table_3.pdf]

Supplementary Table S3. Changes from study baseline in mUFC, LNSC, and random serum cortisol levels (extended evaluation population)

Parameter	Study Baseline		Change From Study Baseline to Month 6			Change From Study Baseline to Month 9			Change From Study Baseline to Month 12		
	**Mean (SD)**	***n***	**Mean (SD)**	***n***	***P* value***	**Mean (SD)**	***n***	***P* value***	**Mean (SD)**	***n***	***P* value***
mUFC, nmol/24h	528.1 (438.8)	60	–397.9 (461.4)	54	<0.0001	–383.9 (421.7)	49	<0.0001	–328.1 (475.6)	44	<0.0001
LNSC, nmol/L	11.7 (17.1)	58	–5.7 (18.5)	54	0.028	–4.9 (21.7)	47	0.131	–2.9 (24.2)	37	0.475
Random serum cortisol, nmol/L[†]	525.2 (150.0)	27	–71.5 (189.3)	22	0.091	–39.8 (165.8)	23	0.262	–74.3 (225.3)	16	0.207

*Two-sided *P* value from the paired t-test performed on the change from study baseline to Months 6, 9, and 12.

[†]No restriction on the clock time samples were collected.

LNSC, late-night salivary cortisol; mUFC, mean urinary free cortisol.
