# Supplementary material for: Levoketoconazole treatment in endogenous Cushing’s syndrome: extended evaluation of clinical, biochemical, and radiologic outcomes
Source: Eur J Endocrinol. 2022 Oct 17;187(6):859–71. doi: 10.1530/EJE-22-0506 (PMC9716395; doi:10.1530/EJE-22-0506)
Supplement: Supplementary Table S4. Changes from Month 6 in mUFC and LNSC (extended evaluation population) [file supplementary_table_4.pdf]

Supplementary Table S4. Changes from Month 6 in mUFC and LNSC (extended evaluation population)

Parameter	Month 6		Change From Month 6 to Month 9		*P* value*	Change From Month 6 to Month 12		*P* value*
	**Mean (SD)**	***n***	**Mean (SD)**	***n***		**Mean (SD)**	***n***	
mUFC, nmol/24h	147.4 (106.4)	54	31.2 (151.5)	44	0.1794	46.3 (135.4)	39	0.0393
LNSC, nmol/L	6.1 (5.5)	55	1.3 (10.2)	44	0.3920	5.4 (14.2)	36	0.0271

*Two-sided *P* value from the paired t-test performed on the change from study baseline to Months 6, 9, and 12.

LNSC, late-night salivary cortisol; mUFC, mean urinary free cortisol.
