# Supplementary material for: Levoketoconazole treatment in endogenous Cushing’s syndrome: extended evaluation of clinical, biochemical, and radiologic outcomes
Source: Eur J Endocrinol. 2022 Oct 17;187(6):859–71. doi: 10.1530/EJE-22-0506 (PMC9716395; doi:10.1530/EJE-22-0506)
Supplement: Supplementary Table S5. mUFC concentrations in patients who had prior radiation therapy at Months 6, 9, and 12 (extended evaluation population) [file supplementary_table_5.pdf]

Supplementary Table S5. mUFC concentrations in patients who had prior radiation therapy at Months 6, 9, and 12 (extended evaluation population)

Patient*	mUFC					
	Month 6		Month 9		Month 12	
	nmol/24 h	×ULN	nmol/24 h	×ULN	nmol/24 h	×ULN
1	210.5	1.5	96.0	0.7	277.4	2.0
2	161.3	1.2	215.4	1.6	195.1	1.4
3	160.2	1.2	129.4	0.9	102.3	0.7
4	129.8	0.9	181.1	1.3	164.7	1.2
5	109.7	0.8	134.3	1.0	216.6	1.6
6	46.6	0.3	48.4	0.4	37	0.3

ULN for UFC = 138 nmol/24 h.

*One patient did not have mUFC values at Months 6, 9, and 12.

mUFC, mean urinary free cortisol; UFC, urinary free cortisol; ULN, upper limit of normal.
