# Supplementary material for: Levoketoconazole treatment in endogenous Cushing’s syndrome: extended evaluation of clinical, biochemical, and radiologic outcomes
Source: Eur J Endocrinol. 2022 Oct 17;187(6):859–71. doi: 10.1530/EJE-22-0506 (PMC9716395; doi:10.1530/EJE-22-0506)
Supplement: Supplementary Table S6. ACTH levels in patients with Cushing’s disease based on mUFC normalization status at Month 12 (extended evaluation population) [file supplementary_table_6.pdf]

Supplementary Table S6. ACTH levels in patients with Cushing's disease based on mUFC normalization status at Month 12 (extended evaluation population)

	**Patients With mUFC Normalization at Month 12**		**Patients Without mUFC Normalization at Month 12**	
	n	**Mean (SD), ACTH ×ULN**	***n***	**Mean (SD), ACTH ×ULN**
Month 6	14	3.0 (2.1)	18	2.7 (2.2)
Month 9	15	3.3 (2.5)	21	2.5 (1.8)
Month 12	15	2.7 (2.2)	20	2.7 (2.0)

ACTH, adrenocorticotropic hormone; mUFC, mean urinary free cortisol; ULN, upper limit of normal.
